# Dissection of the macrophage response towards infection by the *Leishmania*-viral endosymbiont duo and dynamics of the type I interferon response

**DOI:** 10.3389/fcimb.2022.941888

**Published:** 2022-08-04

**Authors:** Amel Bekkar, Nathalie Isorce, Tiia Snäkä, Stéphanie Claudinot, Chantal Desponds, Dmitry Kopelyanskiy, Florence Prével, Marta Reverte, Ioannis Xenarios, Nicolas Fasel, Filipa Teixeira

**Affiliations:** ^1^ Department of Immunobiology, University of Lausanne, Epalinges, Switzerland; ^2^ Agora Center, Center Hospitalier Universitaire (CHUV), Lausanne, Switzerland; ^3^ Center for Integrative Genomics, University of Lausanne, Lausanne, Switzerland

**Keywords:** *Leishmania* RNA virus 1 (LRV1), macrophage, RNA sequencing (RNA-Seq), type I interferon (IFN), weighted gene coexpression network analysis (WGCNA), interleukin 6 (IL-6), tumor-necrosis factor alpha (TNF-α)

## Abstract

*Leishmania* RNA virus 1 (LRV1) is a double-stranded RNA virus found in some strains of the human protozoan parasite *Leishmania*, the causative agent of leishmaniasis, a neglected tropical disease. Interestingly, the presence of LRV1 inside *Leishmania* constitutes an important virulence factor that worsens the leishmaniasis outcome in a type I interferon (IFN)–dependent manner and contributes to treatment failure. Understanding how macrophages respond toward *Leishmania* alone or in combination with LRV1 as well as the role that type I IFNs may play during infection is fundamental to oversee new therapeutic strategies. To dissect the macrophage response toward infection, RNA sequencing was performed on murine wild-type and *Ifnar*-deficient bone marrow–derived macrophages infected with *Leishmania guyanensis* (*Lgy*) devoid or not of LRV1. Additionally, macrophages were treated with poly I:C (mimetic virus) or with type I IFNs. By implementing a weighted gene correlation network analysis, the groups of genes (modules) with similar expression patterns, for example, functionally related, coregulated, or the members of the same functional pathway, were identified. These modules followed patterns dependent on *Leishmania*, LRV1, or *Leishmania* exacerbated by the presence of LRV1. Not only the visualization of how individual genes were embedded to form modules but also how different modules were related to each other were observed. Thus, in the context of the observed hyperinflammatory phenotype associated to the presence of LRV1, it was noted that the biomarkers tumor-necrosis factor α (TNF-α) and the interleukin 6 (IL-6) belonged to different modules and that their regulating specific Src-family kinases were segregated oppositely. In addition, this network approach revealed the strong and sustained effect of LRV1 on the macrophage response and genes that had an early, late, or sustained impact during infection, uncovering the dynamics of the IFN response. Overall, this study contributed to shed light and dissect the intricate macrophage response toward infection by the *Leishmania*-LRV1 duo and revealed the crosstalk between modules made of coregulated genes and provided a new resource that can be further explored to study the impact of *Leishmania* on the macrophage response.

## Introduction

Macrophages, one of the first-line defenders of our immune system, are implicated in many processes ranging from physiological to pathological. One of such processes includes the recognition and ultimately elimination of pathogens. In order to achieve that, macrophages employ a plethora of mechanisms such as oxidative burst, inflammation, or antigen presentation (Shapouri-Moghaddam et al., 2018; Rossi and Fasel, 2018a; Ferrari, 2019; Sheu and Hoffmann, 2022; Reverte et al., 2022). However, these responses are not always straightforward nor unidirectional as pathogens use different strategies to hijack the macrophage machinery and ultimately exploit it to their advantage. This is particularly true for intracellular pathogens such as *Leishmania* that have evolved over thousands of years to counteract the microbicidal power of macrophages (Gupta et al., 2013; Rossi and Fasel, 2018a; Reverte et al., 2022).

*Leishmania* are medically relevant protozoan parasites that cause leishmaniasis, a neglected tropical disease that can manifest depending on the infecting species and the immune status of the host. These manifestations can range from skin ulcers to the disfiguration of the nasopharyngeal area to even potentially lethal forms if left untreated (Pearson and Anastacio de Queiroz, 1996; Scorza et al., 2017; Burza et al., 2018). Although faced with a hostile environment when phagocytosed by macrophages, *Leishmania* have evolved mechanisms to subvert the macrophage response and ultimately prevail. *Leishmania* can, for example, induce the SOCS (suppressor of cytokine signaling) proteins that are negatively regulating the JAK/STAT pathway and the cytokine release leading to the impairment of the crosstalk of the macrophage with the T cell, resulting in the decrease of interleukin 12 (IL-12) and interferon (IFN) gamma (IFN-γ) productions (Chandrakar et al., 2020). Moreover, it has been shown that *Leishmania* are able to block AIF1 (allograft inflammatory factor-1) expression in macrophages and degrade CXCL1 to inhibit the pro-inflammatory responses and the recruitment of neutrophils, respectively (Yorek et al., 2019; da Silva et al., 2021). Parasites can also inhibit the inflammasome through the upregulation of the A20 protein (Hartley et al., 2018; Zamboni and Sacks, 2019) or interfere with the oxidative stress response (Reverte et al., 2021; Reverte et al., 2022).

*Leishmania* RNA virus 1 (LRV1) is a double-stranded RNA (dsRNA) virus of the *Totiviridae* family living endosymbiotically in some strains of *Leishmania* (Tarr et al., 1988; Stuart et al., 1992; Zamora et al., 2000; Hartley et al., 2012). The presence of LRV1 could contribute to the exacerbation and chronicity of leishmaniasis, the development of destructive metastatic lesions, and even treatment failure (Ives et al., 2011; Cantanhede et al., 2015; Adaui et al., 2016; Bourreau et al., 2016; Hartley et al., 2016). The presence of LRV1 inside *Leishmania* results in increased macrophage survival in a mechanism dependent on Akt (Eren et al., 2016); an increased production of pro-inflammatory cytokines and chemokines such as interleukin 6 (IL-6), tumor-necrosis factor α (TNF-α), interleukin 17 (IL-17), type I IFNs (IFN-α and IFN-β) (Ives et al., 2011; Hartley et al., 2016; Rossi et al., 2017); and the inhibition of inflammasomes *via* NLRP3 (Hartley et al., 2018). This immunophenotype depends on the recognition of LRV1 by the endosomal Toll-like receptor 3 (TLR3) (Ives et al., 2011; Olivier and Zamboni, 2020) and the activation of its downstream type I IFN signaling pathways (Rossi et al., 2017). Interestingly, despite the inflammatory environment triggered by LRV1, mice are more susceptible to *Leishmania guyanensis* (*Lgy*) bearing LRV1 (from now on *Lgy*LRV1+) than to *Lgy* devoid of the virus (i.e., *Lgy*LRV1-). Interestingly, the same exacerbatory effect is observed when an exogenous virus is coadministered with *Lgy*LRV1-, resulting in an increase in parasite burden and footpad swelling (Rossi et al., 2017) underlining the importance of the systemic production of type I IFN and its signaling pathways for the exacerbation of the affliction outcome. Mice lacking *Ifnar* (*Ifnar^-/-^*), the gene coding for IFNAR, the receptor of type I IFNs, exhibited a similar lesion size and parasitemia when infected with both LRV1+ and LRV1- parasites (Rossi et al., 2017). Additionally, infection with a virus even after the resolution of the *Leishmania* infection has the potential to reactivate the disease caused by *Leishmania* (Rossi et al., 2017), raising concerns on leishmaniasis control and management. Thus, viral coinfections or later exposure to any potential trigger of the type I IFN response can be considered a risk factor for leishmaniasis relapses. Despite the relevance of viral infection in the context of leishmaniasis, not many studies exist on the role of virus coinfection with *Leishmania*, with the exception perhaps of a coinfection with HIV, known to modulate and impair the adaptive immune response and can thus be considered a predictor of a worsened leishmaniasis outcome (Lindoso et al., 2016).

The advent of transcriptomics has contributed to the global comprehension of how macrophages respond toward infection, including infection by *Leishmania*. A large number of genes modulated by *Leishmania* were related to the immune response (pro- and anti-inflammatory), glycolysis, lipid metabolism, biogenesis, and phagocytosis (Fernandes et al., 2016; Aoki et al., 2017; Aoki et al., 2019; Shadab et al., 2019; Restrepo et al., 2021; Reverte et al., 2021; Salloum et al., 2021; Chaparro et al., 2022). While these studies have shed light on a plethora of mechanisms potentially perturbed by *Leishmania*, they have not explored the impact that coexposure to additional agents may have on the immune response mounted toward *Leishmania*. As mentioned above, this point is particularly relevant when macrophages are concomitantly exposed to the *Leishmania* and its endosymbiont, the LRV1 duo. Understanding how the host responds toward infection by *Leishmania* and the contribution of the presence of the virus and its downstream type I IFN response is paramount to design strategies for improving disease prevention, progression, and the outcome. To have a comprehensive and dynamic understanding of the responses mounted by the macrophage toward the *Leishmania*-LRV1 duo as well as to dissect their individual impact, their synergies or their opposing effects, an RNA sequencing (RNA-Seq) analysis was performed. Here, the different cellular pathways affected by the presence of *Leishmania* or a virus were disentangled and the genes driving those pathways were identified. In addition, the pathways exacerbated by the presence of LRV1 inside *Leishmania* that contributed to worsening the disease fallout were spotted. Interestingly, although non-pathogenic for humans, LRV1 had the potential to surpass the effect of *Leishmania* and ultimately drive the immune response of the macrophage. The dynamic and timely nature of the orchestrated macrophage response was demonstrated by the importance of the genes that had an early, late, or sustained response during infection by the *Leishmania*-LRV1 duo.

## Materials and methods

### Animals

Wild-type (WT; C57BL/6JOlaHsd) mice were purchased from Envigo (Gannat, France), *Ifnar^-/-^* (B6.129S2 *Ifnar1^tm1Agt^*/Mmjax) mice were obtained from M. Aguet, Swiss Institute of Experimental Cancer Research, Epalinges, Switzerland. Mice were maintained and bred in GM500 IVC Green Line cages (Tecniplast, Buguggiate, Italy) at the specific pathogen-free (SPF) animal facility at the University of Lausanne (Epalinges site), with a relative humidity 55 ± 10%, 21 ± 2°C, and in 11/13-h dark/light cycles with *ad libitum* water (local acidified and autoclaved water) and food (Kliba Nafag or Safe). Cardboard or plastic tunnels and igloos, as well as paper tissues, were supplied as enrichment. Bone marrow was extracted from 7-week-old female WT mice (RNA-Seq dataset#1), 7- to 10-week-old female WT mice (RNA-Seq dataset#2), and 9-week- old female *Ifnar^-/-^* mice (RNA-Seq dataset#2) (**Table 1**). Experiments were performed according to the ethical guidelines set out by the Swiss Federal Food Safety and Veterinary Office (FSVO), and procedures were approved by the Veterinary Commission of the Canton de Vaud (SCAV, Switzerland) under the authorization numbers 2113.3.

**Table 1:** Summary table of RNA sequencing samples used for the analysis.

RNA-Seq datasets	Dataset#2		Dataset#1
**Genotypes**	WT	*Ifnar* ^-/-^ (*Ifnar1* ^-/-^)	WT
**Ages of mice**	7–10 weeks old	9 weeks old	7 weeks old
**Number of mice (quality control)**	4	4	5
**Number of mice (RNA-Seq)**	3	3	3
**Conditions** **(treatment/infection)**	Medium, *Lgy*LRV1-, *Lgy*LRV1+, poly I:C	Medium, *Lgy*LRV1-, *Lgy*LRV1+	*Lgy*LRV1-, *Lgy*LRV1+, *Lgy*LRV1- + IFN-α, *Lgy*LRV1- + IFN-β
**Multiplicity of infection (MOI)**	MOI 5		MOI 3
**Times posttreatment/infection**	8 and 24 h		

Table explaining the differences and the similarities between the two datasets of samples analyzed in this study. The factors represented are the experimental steps preceding the final sequencing of the RNA. Two different genotypes, wild type (WT) and Ifnar^-/-^, at two time points p.i. (8 and 24 h) were used. Both genotypes were treated with medium, LgyLRV1+ parasites, LgyLRV1- parasites, or poly I:C. WT was treated in addition to LgyLRV1- parasites + IFN-α, or LgyLRV1- parasites + IFN-β.

### Parasites

The study was performed using lines of *Leishmania guyanensis* (MHOM/BR/78/M4147), named *Lgy*LRV1+ and *Lgy*LRV1-, bearing LRV1 or not, respectively (Kuhlmann et al., 2017). Both parasites were recovered from footpads of infected C57BL/6 mice and not kept for longer than six passages. *Leishmania* strains were cultured in a complete Schneider’s medium, containing a Schneider’s Drosophila Medium (Gibco, Thermo Fischer Reinach, Switzerland) supplemented with 20% heat-inactivated fetal bovine serum (FBS; Gibco), 1% penicillin–streptomycin solution (BioConcept, Allschwill, Switzerland) (i.e., 100 IU/ml and 100 µg/ml, respectively), 2% 4-(2-hydroxyethyl)-1-piperazineethanesulfonic acid (HEPES) (BioConcept) (i.e., 20 mM), hemin–folate solution (prepared from Porcine Hemin, Sigma-Aldrich and Folic Acid, Fluka) (5 and 10 µg/ml, respectively), and 6-Biopterin (Sigma-Aldrich, Buchs, Switzerland) (0.6 µg/ml). They were maintained at 26°C and diluted every week. Six-day-cultured (stationary phase) parasites were used for macrophage infection.

### Bone marrow–derived macrophages

Macrophages were derived from the bone marrow of C57BL/6 mice (WT or *Ifnar^-/-^*). The mice of each genotype were sacrificed, and hind leg bones were collected (both tibia and femur) and kept in the Dulbecco’s modified Eagle’s medium (DMEM; Gibco) on ice. The same following procedure was applied to each mouse. After cleaning the bones with 70% ethanol and the DMEM, the bone marrow was flushed out with the complete DMEM, containing DMEM, supplemented with 10% heat-inactivated FBS (Gibco), 1% penicillin–streptomycin solution (BioConcept) (i.e., 100 UI/ml and 100 µg/ml, respectively), and 1% HEPES (BioConcept) (i.e., 10 mM). The bone marrow suspension was then passed through a 40-µm cell strainer (Corning) and centrifuged for 10 min, at 1,500 rpm (453 × *g*), at 4°C. The cell pellet was resuspended with the complete DMEM medium containing 50-ng/ml recombinant mouse macrophage colony stimulating factor (M-CSF, ImmunoTools). Approximately 10 ml were distributed per Sterilin Petri Dish (Thermo Scientific), and six plates were prepared. After 3 days in culture at 37°C and 5% CO_2_, 5 ml of the complete DMEM medium containing 50-ng/ml M-CSF were added per plate. The cells were differentiated into macrophages for three additional days, that is, 6 days total, at 37°C and 5% CO_2_. After these 6 days, the bone marrow–derived macrophages were detached with 1X Dulbecco's phosphate-buffered saline (DPBS) (Gibco) containing 5 mM ethylenediamine tetraacetic acid (EDTA), washed and resuspended with the complete DMEM medium. The cells were then counted and adjusted to 2.6 million cells per milliliter. They were respectively plated in 12-well plates (TPP) with 1.95 million cells per well. They were finally put back in culture at 37°C and 5% CO_2_ for 1 day.

### Macrophage infection and treatments

The day after their plating, stationary-phase *Leishmania* promastigotes were centrifuged, washed with 1X DPBS, resuspended with a complete DMEM medium, counted, and adjusted to the concentration required. Murine bone marrow–derived macrophages (BMDMs) were infected with the *Leishmania* at multiplicity of infection (MOI) of three and five parasites per macrophage, in the first and the second RNA-Seq datasets, respectively. Cells were also alternatively treated with some synthetic TLR agonist or cotreated with recombinant murine type I IFNs. Poly I:C (HMW), a TLR3 agonist (mimicking dsRNA), was obtained from *In vivo*Gen and used at 2 µg/ml. Murine recombinant IFN-α and IFN-β were used each at 1,000 IU/ml (CellScience). *Lgy*LRV1-infected BMDMs were treated or not with type I IFNs at 6 h postinfection (p.i.). BMDMs in the complete DMEM alone were used as a control. Cells were incubated at 35°C and 5% CO_2_ until 8 and 24 h p.i. or posttreatment (**Table 1**).

### RNA isolation and selection

At 8 and 24 h posttreatment, BMDMs cultured in 12-well plates were cleared out of supernatants and lysed with 350 µl of the RLT buffer (Qiagen) supplemented with 40-mM DTT (Dithiothreitol) for RNA extraction. Plates were then frozen at -80°C until the RNA purification. The RNA samples were purified with the RNeasy Plus Mini Kit (Qiagen) following the manufacturer’s instructions. Purified RNA was eluted with 30 µl of RNase-free water (Qiagen), and their RNA concentrations were measured with a spectrophotometer (NanoDrop, ThermoFisher Scientific). The quality of the RNA samples was then evaluated with a Fragment Analyzer (Agilent) at the Lausanne Genomic Technologies Facility (GTF) for both RNA-Seq datasets. For the dataset#2, the RNA quantification was performed with a fluorimetric method (Ribogreen; ThermoFisher Scientific) by the GTF according to the manufacturer recommendations, then used to prepare the RNA dilutions for the libraries. While for the dataset#1, the total integrated concentration (TIC) values given by the Fragment Analyzer were directly used to prepare the RNA dilutions for the libraries. The RNA samples diluted for the libraries were the ones that have an RNA quality number (RQN) higher than 6 (for dataset#2) and 7 (for dataset#1) and from 3 mice out of 4 and 5, respectively. The samples were diluted in 96-well plates (Labgene Scientific). Then, from these plates, the libraries and the sequencing were performed at the GTF.

### RNA sequencing data processing

Purity-filtered reads were adapters and quality trimmed with Cutadapt [v. 1.8 (Martin, 2011)]. Reads matching to ribosomal RNA sequences were removed with fastq_screen (v. 0.9.3 for dataset#1 and v. 0.11.1 for dataset#2). The remaining reads were further filtered for low complexity with reaper [v. 15-065, (Davis et al., 2013)]. Reads were aligned against the Mus musculus.GRCm38.86 genome using STAR [v. 2.5.2b, (Dobin et al., 2013)] for dataset#1 and the Mus musculus.GRCm38.92 genome using STAR [v. 2.5.3a, (Dobin et al., 2013)] for dataset#2. The number of read counts per gene locus was summarized with htseq-count [v. 0.6.1 for dataset#1 and v. 0.9.1 for dataset#2, (Anders et al., 2015)] using Mus musculus.GRCm38.86 gene annotation for dataset#1 and Mus musculus.GRCm38.92 gene annotation for dataset#2. The quality of the RNA-seq data alignment was assessed using RSeQC [v. 2.3.7, (Wang et al., 2012)]. Reads were also aligned to the Mus musculus.GRCm38.86 transcriptome using STAR [v. 2.5.2b, (Dobin et al., 2013)] for dataset#1 and to the Mus musculus.GRCm38.92 transcriptome using STAR [v. 2.5.3a, (Dobin et al., 2013)] for dataset#2. The estimation of the isoform abundance was computed using RSEM [v. 1.2.31, (Li and Dewey, 2011)].

### Normalization and data transformation

Statistical analysis was performed for genes independently in the software environment R. Genes with a low number of counts were filtered out according to the rule of one count(s) per million (cpm) in at least one sample. Library sizes were scaled using TMM normalization. Subsequently, the normalized counts were transformed to cpm values, and a log2 transformation was applied, by means of the function cpm with the parameter setting prior.counts = 1 [EdgeR (Robinson et al., 2010)]. For the analysis with *Ifnar^-/-^* and WT genotypes, two sets of data generated separately were merged and this to integrated WT samples treated with IFN-α and IFN-β. To check if this was possible, we plotted the distributions of the log cpm of the shared conditions between the two datasets (*Lgy*LRV1+ and *Lgy*LRV1-). The distributions had similar patterns at 8- and 24-h time points, and the different groups of conditions were well separated (**Supplementary Figure S1**). Given the fact that with WGCNA, we are looking at expression patterns across conditions and not individual gene differential expression, we found this sufficient to take advantage of having more experimental conditions by integrating a dataset with WT treated with IFN-α and IFN-β.

### Weighted gene coexpression network analysis and downstream bioinformatics analysis

Weighted gene coexpression network analysis (WGCNA) (Langfelder and Horvath, 2008) was performed on normalized RNA-Seq data in R (package WGCNA 1.69). For each analysis, an adjacency matrix was calculated to construct a signed hybrid coexpression network using Spearman correlation. A sequence of soft-thresholding powers was tested to reach a free-topology network with a relatively low mean connectivity, and the following thresholds were chosen for each analysis: 14 for WT at 8 h p.i., 9 for WT at 24 h p.i., 7 for WT + *Ifnar^-/-^* at 8 h p.i., and 16 for WT + *Ifnar^-/-^* at 24 h p.i. A topological overlap matrix (TOM) was then calculated from the adjacency matrix, converted to distance and clustered by hierarchical clustering using average linkage clustering. Modules were identified by a dynamic tree cut with a minimum module size = 20. Module eigengenes (MEs) that are the first principal component of the module were calculated, and similar modules were merged together using an ME distance threshold of 0.08 for WT at 8 h p.i., 0 for WT at 24 h p.i., 0.18 for WT + *Ifnar^-/-^* at 8 h p.i., and 0.08 for WT + *Ifnar^-/-^* at 24 h p.i. (**Supplementary Figures S2–S5**). kWithin that is the intramodular connectivity and kTotal, the whole network connectivity of each gene, were calculated with the WGCNA package. The relationship of MEs with the infection status (independent variable) was assessed with a regression analysis on MEs (dependent variable) for each module separately. The WT Medium condition was used as a reference (intercept). Adjusted R-squared and *p*-values were used to assess the model performance. ME average predictions were plotted as a heatmap. Adjusted R-squared (R-squared adjusted for the number of predictors in the model) that is a statistical measure that represents the proportion of the variance for a dependent variable (expression) that is explained by an independent variable (infection groups) was calculated by fitting a linear model for each gene expression value. Gene-adjusted R-squared was then plotted against kTotal values. Gene Ontology (GO) enrichment analysis was performed for gene coexpression modules against GO categories using the topGO R package (topGO 2.26.0) (Alexa and Rahnenfuhrer, 2016) and GO database (07.2019)  (Ashburner et al., 2000; Gene Ontology Consortium, 2021). Closeness centrality (CC) (Freeman, 1978) in the coexpression network was calculated for each gene at 8- and 24-h time points using the Networkx Python package (Networkx 2.5). A density map was plotted of CC at 8 h against CC at 24 h. Network visualization was performed with Cytoscape.

## Results

### The global network analysis of wild-type-infected macrophages highlighted modules correlated to either *Leishmania*, LRV1 or to Leishmania but further exacerbated by LRV1

**Figure 1:**
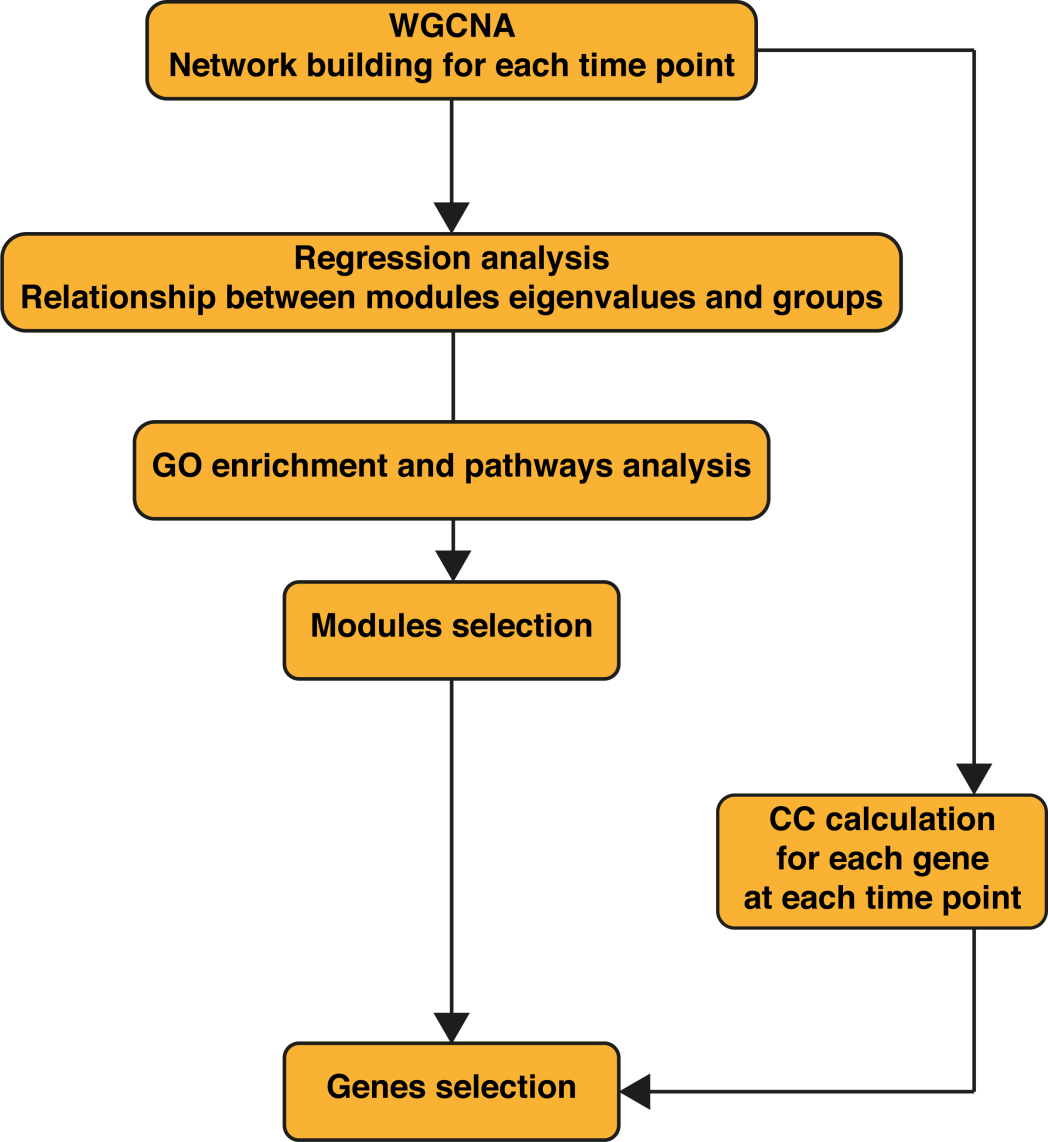
Workflow of the bioinformatics analysis. WGCNA was performed first on the wild-type (WT) samples alone, then on WT + *Ifnar^-/-^* samples merged. Regression analysis was performed on obtained modules to assess their relationship to phenotypes (infection groups). Gene Ontology (GO) enrichment was performed for each module. WT + *Ifnar^-/-^* network resulting from weighted gene correlation network analysis (WGCNA) was used to calculate the closeness centrality (CC) of genes at 8 and 24 h postinfection (p.i.).

**Figure 2:**
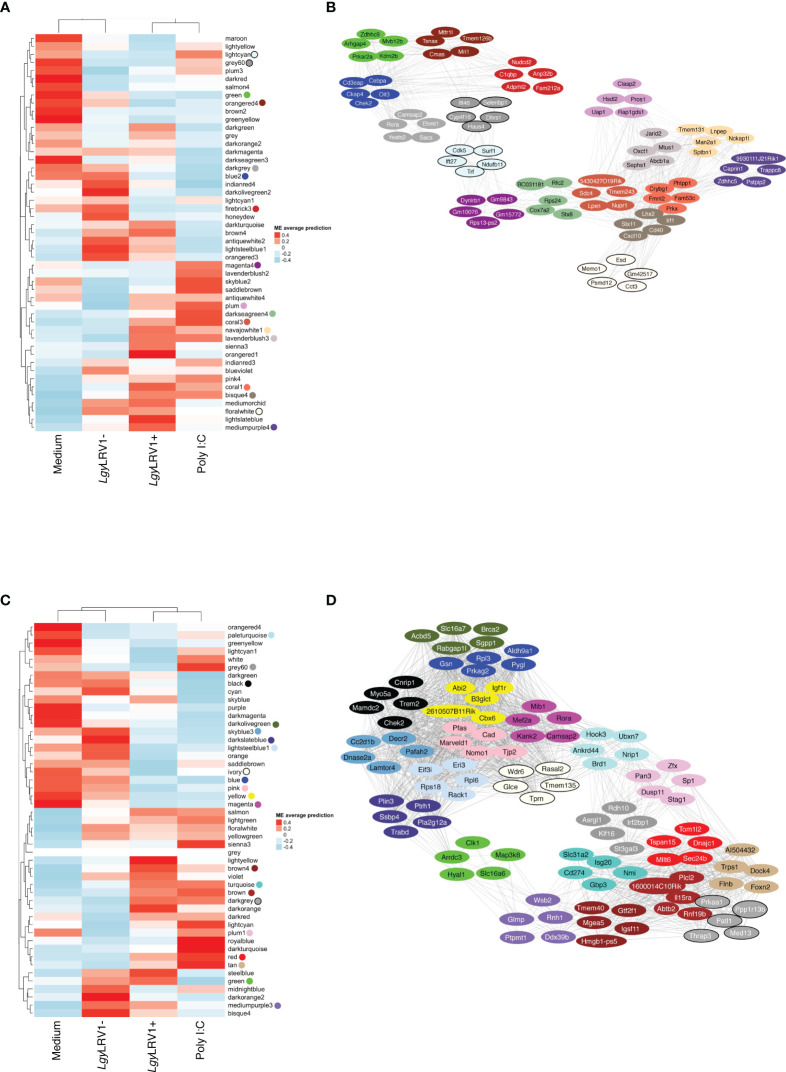
The global network analysis of WT-infected macrophages highlights modules and key pathways associated to *Leishmania* and to *Leishmania* RNA virus 1 (LRV1). **(A)** Heatmap of the average predictions of the fitted linear model on each module eigengene (ME) at the 8-h time point. **(B)** Network generated from selected modules at the 8-h time point associated with *Leishmania* infection (*Lgy*LRV1+, *Lgy*LRV1-), virus (*Lgy*LRV1+, poly I:C), and “exacerbatory” modules. Only the top five highest connected genes were selected. Node colors indicate the module color they belong to. Edges between genes indicate the correlation between genes. **(C)** Heatmap of average predictions of the fitted linear model on each ME at the 24-h time point. **(D)** Network generated from selected modules at the 24-h time point associated with *Leishmania* infection (*Lgy*LRV1+, *Lgy*LRV1-), virus (*Lgy*LRV1+, poly I:C), and “exacerbatory” modules. Only the top five highest connected genes were selected. Node colors indicate the module color they belong to. Edges between genes indicate the correlation between genes.

To design appropriate strategies to combat leishmaniasis and understand its clinical presentations it was important to unravel the influence of *Leishmania* or LRV1 on the response mounted by the macrophage upon infection by *Lgy*LRV1+. To this end, transcriptome profiling using RNA-Seq was performed to identify global changes in the WT murine macrophage infected with *Lgy*, carrying or not LRV1, defined as LRV1+ or LRV1-, at 8 and 24 h p.i. As a control, cells were left non-infected (Medium) or treated with polyinosinic-polycytidylic acid (poly I:C), a TLR3 agonist, mimicking solely the effect of dsRNA viruses such as LRV1. Of the 36,487 genes annotated for *Mus musculus* (McGarvey et al., 2015), 12,651 and 12,594 genes were detected in this study at 8 and 24 h p.i., respectively, and used for downstream analysis as shown in **Figure 1**. First, a weighted gene coexpression network analysis (WGCNA) was performed. WGCNA is a systems biology approach that relies on the hypothesis that genes with similar expression patterns may be functionally related, coregulated, or members of the same pathway. Following this approach, a gene–gene similarity network was constructed allowing to identify highly correlated genes that cluster into modules. The expression profile of the module genes was summarized by the ME that is the first principal component of its expression. Upon identification of the modules a regression analysis was performed on modules eigengenes and the average prediction was calculated to identify modules that were associated with the different experimental groups (Medium, *Lgy*LRV1+, *Lgy*LRV1- and poly I:C). The intramodular (kWithin) and whole network (kTotal) connectivity were computed for each gene allowing to identify hubs (drivers). Following the WGCNA, 49 modules at 8 h and 49 at 24 h were defined. The different modules are represented as a heatmap based on the average predictions of the regression analysis in the different experimental groups in **Figures 2A, C** (*p*-values shown in **Supplementary Figures S6A, B**).

The blue to red scale speaks for negative to positive associations with a determined condition based on normalized enrichment scores. On top of each heatmap a dendrogram, representing the hierarchical clustering of the data, is shown. Interestingly, although *Leishmania* is the common denominator between *Lgy*LRV1+ and the *Lgy*LRV1- groups, *Lgy*LRV1- clusters correlated closer to the group left untreated (mentioned as “Medium” in **Figure 2**) while *Lgy*LRV1+ clusters correlated closer to the poly I:C group both at 8 and 24 h posttreatment. This observation demonstrates that the presence of LRV1 within *Leishmania* triggers a drastic macrophage response, shifting its profile closer to the response triggered solely by a virus than to that of *Leishmania* alone.

Based on the analysis of the heatmaps, the modules were defined as either *Leishmania*- or LRV1 dependent. Comparatively to the medium condition, the LRV1-modulated group encompassed modules exclusively up- or downregulated by *Lgy*LRV1+ and poly I:C but not modulated by *Leishmania* devoid of LRV1 (i.e., *Lgy*LRV1-). On the other hand, the *Leishmania*-modulated group was characterized by modules similarly regulated by *Lgy*LRV1- and *Lgy*LRV1+ comparatively to medium, but not by poly I:C. Among the 49 modules at each time point, and 5 modules were found to be associated with *Leishmania* at 8 and 24 h p.i., respectively, and 6 and 9 modules were found to be associated with the presence of LRV1 at 8 and 24 h p.i., respectively. Thus, the modules selected for further analysis due to their association with *Leishmania* were lightcyan (245 genes), grey60 (255 genes), magenta4 (70 genes), floralwhite (112 genes) at 8 h p.i. and mediumpurple3 (116 genes), green (493 genes), plum1 (134 genes), grey60 (185 genes), paleturquoise (148 genes) at 24 h p.i. The modules selected due to their association to LRV1 were blue2 (725 genes), firebrick3 (159 genes), coral3 (644 genes), navajowhite1 (130 genes), lavenderblush3 (145 genes), plum (83 genes) at 8 h p.i. and black (345 genes), skyblue3 (135 genes), lightsteelblue1 (116 genes), blue (1207 genes), brown (1137 genes), darkgrey (168 genes), red (352 genes), tan (237 genes), darkslateblue (86 genes) at 24 h p.i. Given the known exacerbatory role that LRV1 has on the leishmaniasis outcome (Ives et al., 2011; Hartley et al., 2012; Cantanhede et al., 2015; Rossi et al., 2017) another category of modules was defined as “exacerbatory”. These so-called “exacerbatory” modules are, to a certain extent modified by *Lgy*LRV1-, but the presence of LRV1 in *Lgy*LRV1+ further exacerbates this response in a similar direction as that observed with the poly I:C treatment. At 8 h p.i., there were 7 “exacerbatory” modules highlighted: green (1,159 genes), orangered4 (435 genes), coral1 (744 genes), darkseagreen4 (344 genes), bisque4 (1,129 genes), darkgrey (884 genes), and mediumpurple4 (158 genes). At 24 h p.i., 6 “exacerbatory” modules: darkolivegreen (145 genes), pink (294 genes), yellow (639 genes), magenta (287 genes), brown4 (99 genes), and ivory (111 genes) were identified. (For the whole lists of genes with their module membership and their connectivity at each time point, see **Supplementary Table S1**).

Based on the above-mentioned selection of modules, the presence of *Leishmania* lead to a concerted modulation of 682 genes at 8 h p.i. and 1,076 genes at 24 h p.i. representing 5.4% and 8.5%, respectively, of the total genes identified. On the other hand, the presence of LRV1 impacted 1,886 genes at 8 h p.i. and 3,783 genes at 24 h p.i. representing 14.9% and 30% of the total amount of genes identified at 8 and 24 h p.i., respectively. As for the “exacerbatory” modules, they comprise a large proportion of the genes with a synchronized modulation, 4853 and 1575 genes representing 38.4% and 12.5% of the genes identified at 8 and 24 h p.i., respectively. This approach gave us a general overview of the macrophage response toward *Leishmania*, LRV1, or the combination of both agents. The results support the importance of the modulation of the macrophage response by LRV1 both at 8 and 24 h p.i.

### The global network analysis of wild-type-infected macrophages highlighted key pathways associated to *Leishmania* and to LRV1

Using the WGCNA groups of genes with a similar expression were identified and classified into modules; however, to shed light into the biological processes that may be present in each of the modules selected above, a GO enrichment analysis using the topGO R package (topGO 2.26.0) (Alexa and Rahnenfuhrer, 2016) and the GO database (Ashburner et al., 2000; Gene Ontology Consortium, 2021) was performed. The aim was to identify annotated functions overrepresented in the modules. Among the biological process (BP) category of the GO terms identified, a curation of the GOs were conducted and GO terms related to either clearly different types of cells or tissues, or even to completely distinct organs or organisms were removed. The 5 most statistically significant terms (with *p*-values strictly lower than 0.01) within a module are shown in **Supplementary Table S2**. The fact that WGCNA analysis resulted in modules enriched for biologically important processes related to infection, including an innate immune response (found in the 8h_coral3 module) and a positive regulation of interleukin-6 production (found in the 8h_bisque4 module), suggest that these modules are a robust feature of the molecular architecture of *Leishmania* and LRV1 infection. Modules associated to *Leishmania* were enriched in terms such as the positive regulation of telomere capping, RNA secondary structure unwinding, purine deoxyribonucleotide, and glutathione metabolic process in the grey60, floralwhite, lightcyan, and magenta4 modules, respectively, at 8 h p.i. (**Supplementary Table S2**). Moreover, at 24 h p.i., the *Leishmania*-associated modules were enriched in terms such as positive regulation of receptor internalization and asymmetric protein localization in mediumpurple3 and plum1, respectively, and the regulation of transcription, negative regulation of focal adhesion assembly, and cellular response to acid chemical in paleturquoise, grey60 and green, respectively (**Supplementary Table S2**). Modules associated with LRV1 at 8 h p.i. were enriched in pathways related to tRNA methylation, transcription, translation, GTPase activity, and DNA replication, in the blue2, firebrick3, navajowhite1, and lavenderblush3 modules at 8 h p.i., whereas coral3 and plum were enriched in the immune response (for example, innate immune response and a positive regulation of acute inflammatory response) and phagocytosis pathways. Comparatively, LRV1-associated modules at 24 h p.i., such as skyblue3, blue, lightsteelblue1, darkgrey, and tan, were enriched in pathways related to methylation, translation, and transcription processes, whereas the black, brown, red, tan, and darkslateblue modules were enriched in phagocytosis, cytokine production, and the oxidation–reduction process (**Supplementary Table S2**). On the other hand, the “exacerbatory” modules were enriched in terms such as the RNA biosynthetic process, oxidation–reduction process and mitosis-related pathways in green, orangered4 and darkseagreen4; and NF-kappaB signaling and cytokine production in coral1 and bisque4 at 8 h p.i. Furthermore, the “exacerbatory” modules at 24 h p.i. were enriched in DNA replication, transcription, and macrophage regulation in the darkolivegreen, yellow, magenta, and brown4 modules (**Supplementary Table S2**).

### Identification of hub genes and network analysis provided insight on how the different modules may interact

In each module, the most central genes, that is, the most interconnected genes measured by their intramodular connectivity, that is, its kWithin, can be further identified as hub genes. Due to their high connectivity, hub genes are thus considered as functionally important genes that are most likely to drive the group of genes and thus the biological processes present within each module. The top 1% of genes with the highest kWithin are shown in **Table 2** for each of the modules selected. In order to visualize the interaction between the *Leishmania* virus and “exacerbatory” modules, the five genes with the highest connectivity for each module (i.e., with the highest kWithin) are displayed as a network for 8 and 24 h p.i. in **Figures 2B, D**. The five top driver genes associated to *Leishmania* were *Ift27*, *Cdk5*, *Surf1*, *Trf*, *Ndufb11* (lightcyan), *Ift46*, *Selenbp1*, *Cyp4f16*, *Dhrs1*, *Haus4* (grey60), *Dynlrb1*, *Gm9843*, *Gm10076*, *Rps13-ps2*, *Gm15772* (magenta4), *Memo1*, *Esd*, *Gm42517*, *Psmd12*, *Cct3* (floralwhite) at 8 h p.i.; and *Ddx39b*, *Ptpmt1*, *Rnh1*, *Glmp*, *Wsb2* (mediumpurple3), *Arrdc3*, *Clk1*, *Map3k8*, *Hyal1*, *Slc16a6* (green), *Zfx*, *Sp1*, *Stag1*, *Dusp11*, *Pan3* (plum1), *St3gal3*, *Klf16*, *Asrgl1*, *Rdh10*, *Irf2bp1* (grey60), *Hook3*, *Ubxn7*, *Ankrd44*, *Brd1*, *Nrip1* (paleturquoise) at 24 h p.i. The five top driver genes inside the modules selected due to their association to LRV1 were *Cd3eap*, *Cebpa*, *Oit3*, *Ckap4*, *Chek2* (blue2), *Nudcd2*, *C1qbp*, *Anp32b*, *Fam212a*, *Adprhl2* (firebrick3), *5430427O19Rik*, *Sdc4*, *Tmem243*, *Nupr1*, *Lpxn* (coral3), *Tmem131*, *Lnpep*, *Man2a1*, *Sptbn1*, *Nckap1l* (navajowhite1), *Jarid2*, *Mtus1*, *Oxct1*, *Abcb1a*, *Sephs1* (lavenderblush3), *Clasp2*, *Pros1*, *Hsdl2*, *Uap1*, *Rap1gds1* (plum) at 8 h p.i.; and *Chek2*, *Myo5a*, *Trem2*, *Cnrip1*, *Mamdc2* (black), *Cc2d1b*, *Dnase2a*, *Decr2*, *Pafah2*, *Lamtor4* (skyblue3), *Rack1*, *Eif3i*, *Rps18*, *Eri3*, *Rpl6* (lightsteelblue1), *Pygl*, *Gsn*, *Prkag2*, *Rpl3*, *Aldh9a1* (blue), *1600014C10Rik*, *Rnf19b*, *Plcl2*, *Abtb2*, *Il15ra* (brown), *Patl1*, *Prkaa1*, *Thrap3*, *Ppp1r13b*, *Med13* (darkgrey), *Tom1l2*, *Mllt6*, *Sec24b*, *Dnajc1*, *Tspan15* (red), *Al504432*, *Trps1*, *Dock4*, *Flnb*, *Foxn2* (tan), *Plin3*, *Pla2g12a*, *Ptrh1*, *Trabd*, *Ssbp4* (darkslateblue) at 24 h p.i. For the “exacerbatory” modules, the 5 top driver genes were: *Arhgap4*, *Zdhhc9*, *Mvb12b*, *Prkar2a*, *Kdm2b* (green), *Tsnax*, *Mtfr1l*, *Tmem126b*, *Cmas*, *Mri1* (orangered4), *Phlpp1*, *Crybg1*, *Fmnl2*, *Fam53c*, *Prkx* (coral1), *BC031181*, *Rfc2*, *Rps24*, *Cox7a2*, *Stx8* (darkseagreen4), *Irf1*, *Lhx2*, *Stx11*, *Cd40*, *Cxcl10* (bisque4), *Rora*, *Camsap2*, *Ehmt1*, *Yeats2*, *Sacs* (darkgrey) and *9930111J21Rik1*, *Caprin1*, *Trappc8*, *Zdhhc5*, *Pstpip2* (mediumpurple4) at 8 h p.i.; and *Acbd5*, *Brca2*, *Slc16a7*, *Sgpp1*, *Rabgap1l* (darkolivegreen), *Nomo1*, *Marveld1*, *Pfas*, *Cad*, *Tjp2* (pink), *Igf1r*, *Abi2*, *B3glct*, *2610507B11Rik*, *Cbx6* (yellow), *Camsap2*, *Kank2*, *Mib1*, *Rora*, *Mef2a* (magenta), *Tmem40*, *Gtf2f1*, *Mgea5*, *Hmgb1-ps5*, *Igsf11* (brown4), *Glce*, *Rasal2*, *Tmem135*, *Wdr6*, *Tprn* (ivory) and *Slc31a2*, *Gpb3*, *Isg20*, *Cd274*, *Nmi* (turquoise) at 24 h p.i. (for the whole lists of genes with their module membership and their connectivity at each time point, see **Supplementary Table S1**).

**Table 2:** The top 1% genes with the highest kWithin of the three groups of modules selected in WT analysis at 8 and 24 h postinfection (p.i).

Time	Group	Module	Top 1% genes
8h	*Leishmania*	lightcyan	*Ndufb11, Ift27, Surf1*
		grey60	*Cyp4f16, Ift46, Haus4*
		magenta4	*Gm9843*
		floralwhite	*Esd*
	LRV1	blue2	*Oit3, Cebpa, Ckap4, Chek2, Cd3eap, Trmt2a, Abcf3, Nat10*
		firebrick3	*Fam212a, C1qbp*
		coral3	*Nupr1, Lpxn, Sdc4, 5430427O19Rik, Tmem243, Stx18, Prpsap1*
		navajowhite1	*Lnpep, Sptbn1*
		lavenderblush3	*Sephs1, Jarid2*
		plum	*Hsdl2*
	Exacerbatory	green	*Prkar2a, Arhgap4, Kdm2b, Zdhhc9, Mvb12b, Cul7, Fads1, Adcy9, Gpd1l, Slc25a36, Manba, Slc25a16*
		orangered4	*Mtfr1l, Tmem126b, Tsnax, Cmas, Mri1*
		coral1	*Fam53c, Prkx, Crybg1, Phlpp1, Fmnl2, Ankrd50, Etv3, Ttc9c*
		darkseagreen4	*Rps24, Cox7a2, Rfc2, BC031181*
		bisque4	*Cd40, Lhx2, Stx11, Cxcl10, Irf1, Phf11b, Gnb4, Ccrl2, Rilpl1, Zufsp, March5, Sp140*
		darkgrey	*Sacs, Camsap2, Yeats2, Rora, Ehmt1, Ndst1, Chd3, Zfp652, Dnmbp*
		mediumpurple4	*Caprin1, 9930111J21Rik1*
24h	*Leishmania*	mediumpurple3	*Rnh1, Ddx39b*
		green	*Map3k8, Arrdc3, Clk1, Hyal1, Slc16a6*
		plum1	*Sp1, Dusp11*
		grey60	*Irf2bp1, Rdh10*
		paleturquoise	*Hook3, Ubxn7*
	LRV1	black	*Mamdc2, Myo5a, Cnrip1, Chek2*
		skyblue3	*Cc2d1b, Lamtor4*
		lightsteelblue1	*Rpl6, Eri3*
		blue	*Aldh9a1, Prkag2, Pygl, Rpl3, Gsn, Cd28, Aplp2, Tnfrsf21, Trim47, Fam134b, Man2a2, Sort1, Adssl1*
		brown	*Plcl2, Rnf19b, 1600014C10Rik, Il15ra, Abtb2, Lcp2, Il15, Nod1, Acsl1, Tnfaip3, Hrh2, Ctsc*
		darkgrey	*Ppp1r13b, Prkaa1*
		red	*Tspan15, Mllt6, Tom1l2, Sec24b*
		tan	*Flnb, Trps1, Dock4*
		darkslateblue	*Plin3*
	Exacerbatory	darkolivegreen	*Acbd5, Rabgap1l*
		pink	*Marveld1, Cad, Tjp2*
		yellow	*Igf1r, B3glct, Cbx6, 2610507B11Rik, Abi2, Adcy9, Grk5*
		magenta	*Rora, Mef2a, Kank2*
		brown4	*Gtf2f1*
		ivory	*Wdr6, Glce*
		turquoise	*Slc31a2, Gpb3, Isg20, Cd274, Nmi, Ogfr, Parp10, H2-Q5, Nr1h3, AW112010, Phf11b, Phf11a, Igtp, Bambi-ps1*

As shown, the top five hub genes of each module were clustered diametrically, depending on whether they were either positively or negatively modulated by a given treatment as shown in **Figure 2B**. Interestingly, at 8 h, the connection between these two extremities was achieved by a module with an exacerbatory phenotype, the 8h_darkseagreen4, enriched in mitosis-related pathways as mentioned before (**Supplementary Table S2**). At 24 h, the connection between modules that were positively or negatively modulated is accomplished by two different branches. Interestingly, the connecting modules at 24 h show different modulations for either *Leishmania* (LRV1+ and LRV1-) or poly I:C (i.e., the molecule used to map virus-dependent responses). On one side, the connection was realized by the 24h_green module consisting of upregulated genes in *Leishmania*-infected macrophages but downregulated in poly I:C-treated cells. As mentioned previously, the 24h_green module was enriched in the cellular response to the acid chemical (**Supplementary Table S2**). On the other side, the link between modules with up- and downregulated genes was achieved by the 24h_grey60 and 24h_plum1 showing both the downregulation of genes associated with *Leishmania* but being either upregulated or non-modulated by poly I:C. As mentioned previously, the 24h_grey60 module was enriched in genes implicated in the negative regulation of focal adhesion assembly and the 24h_plum1 in asymmetric protein localization (**Supplementary Table S2**). The topology of the networks represented by the five top genes from each module suggested the existence of a dependency between the different cascades triggered by *Leishmania* and LRV1 infection.

### The global network analysis of wild-type-infected macrophages highlighted modules with highly connected genes that explained most of the variance of the data (highly adjusted R-squared)

**Figure 3:**
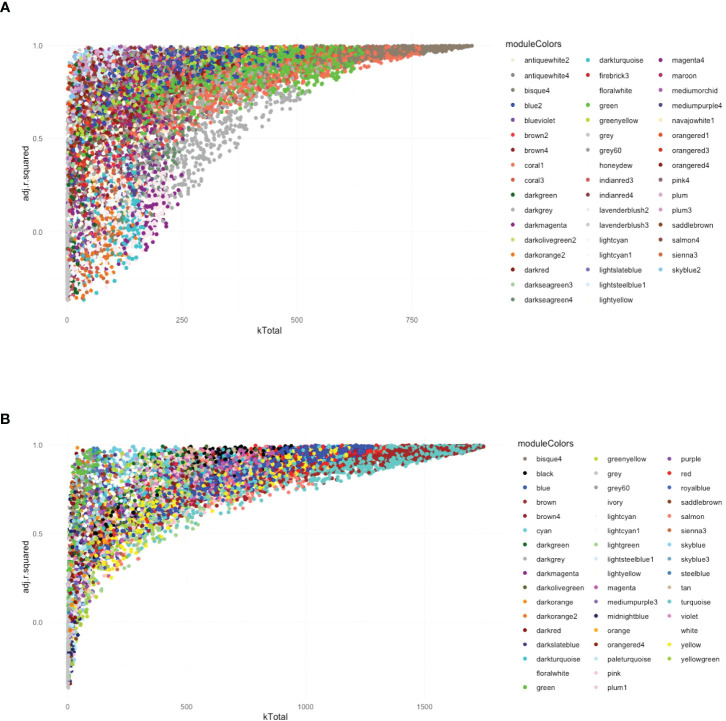
The global network analysis of WT-infected macrophages highlights modules with highly connected genes that explain most of the variance of the data (highly adjusted R-squared). **(A)** Scatter plot of kTotal (whole network connectivity) against adjusted R-squared for all genes in an 8-h network. Genes are colored according to the module (described in **Figure 2**) they belong to. **(B)** Scatterplot of kTotal (whole network connectivity) against adjusted R squared for all genes in the 24 h network. Genes are colored according to the module (described in **Figure 2**) they belong to.

Following the approach described above four, six, and seven modules were found associated to *Leishmania*, LRV1, and the combination of both, respectively, at 8 h p.i., whereas five, nine, and six modules were associated to *Leishmania*, LRV1, and the combination of both, respectively, at 24 h p.i. However, in order to understand which modules had potentially the highest impact on the macrophage transcriptional response, genes were plotted according to their total connectivity (kTotal) and adjusted R-squared (**Figure 3**). This approach allowed us to identify core modules. Thus, the genes present at the tip of the plot belonging to the bisque4, coral1, and green modules at 8 h and brown, turquoise, red, and blue modules at 24 h were likely to be strongly affected by *Leishmania* and LRV1 infection. Interestingly, all modules at 8 h could be considered to have an exacerbatory modulation, while at 24 h, three out of the four modules were virus driven (brown, red, and blue) while turquoise was exacerbatory. This observation indicated that while at early time points, the combination of *Leishmania* and LRV1 had the highest impact on the macrophage response, this effect was mainly achieved later by the presence of LRV1, suggesting that the presence of *Leishmania* at later time points impacts the macrophage response to a much lesser extent than LRV1. Moreover, out of the seven modules present at the tip, five (8h_bisque4; 8h_coral1; 24h_brown; 24h_turquoise and 24h_red; with a total of 1,872 genes at 8 h and 2,810 genes at 24 h) were enriched in pathways associated with the immune response (**Table 3**), highlighting the importance of a coordinated immune response and its overall impact when macrophages were challenged by an external agent. Among these immune-enriched pathways, there were, for example, the GO terms: innate immune response, cellular responses to or the regulations of IFNs or cytokines, defense responses to virus or protozoan (**Table 3**), whereas the other non-immune modules present at the tip were enriched in, for example, the GO terms: RNA biosynthetic and metabolic processes, the regulation of transcription from the RNA polymerase II promoter and oxidation–reduction process (**Table 3**). These results showed how immune response–related genes shape the network topology, emphasizing its impact on the transcriptomic profile of the macrophage and highlighting the strong and sustained effect that LRV1 had on the macrophage response.

**Table 3:** Examples of main Gene Ontology (GO) terms of tip modules in WT analysis (biological process “BP” category and *p*-value < 0.01) at 8 and 24 h p.i.

Time	Module	GO.ID	*p*-value	Term
8h	bisque4	GO:0071346	6.90E-15	cellular response to interferon-gamma
		GO:0035458	3.20E-14	cellular response to interferon-beta
		GO:0051607	1.06E-11	defense response to virus
		GO:0042832	7.51E-11	defense response to protozoan
		GO:0070374	4.53E-10	positive regulation of ERK1 and ERK2 cascade
		GO:0042510	5.12E-07	regulation of tyrosine phosphorylation of Stat1 protein
		GO:0032760	7.69E-07	positive regulation of tumor necrosis factor production
		GO:0050729	1.17E-06	positive regulation of inflammatory response
		GO:0045824	7.87E-06	negative regulation of innate immune response
		GO:0071222	9.11E-06	cellular response to lipopolysaccharide
		GO:0032735	9.38E-06	positive regulation of interleukin-12 production
	coral1	GO:0045087	3.79E-06	innate immune response
		GO:0006468	3.70E-04	protein phosphorylation
		GO:0007250	4.39E-04	activation of NF-kappaB-inducing kinase activity
		GO:2000637	4.39E-04	positive regulation of gene silencing by miRNA
		GO:0035329	6.52E-04	hippo signaling
		GO:0044827	6.77E-04	modulation by host of viral genome replication
	green	GO:0006298	8.05E-04	mismatch repair
		GO:0032774	1.02E-03	RNA biosynthetic process
		GO:0060828	3.22E-03	regulation of canonical Wnt signaling pathway
		GO:0019318	4.62E-03	hexose metabolic process
		GO:0051172	7.21E-03	negative regulation of nitrogen compound metabolic process
24h	brown	GO:0032755	1.42E-07	positive regulation of interleukin-6 production
		GO:0032735	2.21E-06	positive regulation of interleukin-12 production
		GO:0032760	5.16E-06	positive regulation of tumor necrosis factor production
		GO:0070374	1.68E-05	positive regulation of ERK1 and ERK2 cascade
		GO:0032496	1.71E-05	response to lipopolysaccharide
		GO:0042108	2.12E-05	positive regulation of cytokine biosynthetic process
		GO:0006954	1.69E-04	inflammatory response
		GO:0034341	2.65E-04	response to interferon-gamma
		GO:0032693	3.12E-04	negative regulation of interleukin-10 production
		GO:0051607	6.80E-04	defense response to virus
		GO:0034134	1.03E-03	Toll-like receptor 2 signaling pathway
	turquoise	GO:0035458	1.59E-13	cellular response to interferon-beta
		GO:0051607	5.89E-12	defense response to virus
		GO:0071346	2.29E-11	cellular response to interferon-gamma
		GO:0045071	8.86E-11	negative regulation of viral genome replication
		GO:0045087	5.79E-08	innate immune response
		GO:0002474	5.68E-07	antigen processing and presentation of peptide antigen *via* MHC class I
		GO:0060338	2.15E-05	regulation of type I interferon-mediated signaling pathway
		GO:0042832	3.71E-05	defense response to protozoan
		GO:0032388	6.04E-05	positive regulation of intracellular transport
		GO:0070098	1.08E-03	chemokine-mediated signaling pathway
		GO:0045824	1.38E-03	negative regulation of innate immune response
	red	GO:0000122	2.35E-04	negative regulation of transcription from RNA polymerase II promoter
		GO:0032722	5.23E-04	positive regulation of chemokine production
		GO:2000060	2.83E-03	positive regulation of protein ubiquitination involved in ubiquitin-dependent protein catabolic process
		GO:0035690	3.13E-03	cellular response to drug
		GO:0032755	4.38E-03	positive regulation of interleukin-6 production
	blue	GO:0055114	4.51E-06	oxidation–reduction process
		GO:0005975	1.11E-04	carbohydrate metabolic process
		GO:0008203	2.64E-04	cholesterol metabolic process
		GO:0032869	2.69E-04	cellular response to insulin stimulus
		GO:0019369	1.43E-03	arachidonic acid metabolic process

### Type I interferons played a preponderant and central role in the infection mounted by macrophages toward *Lgy*LRV1+

**Figure 4:**
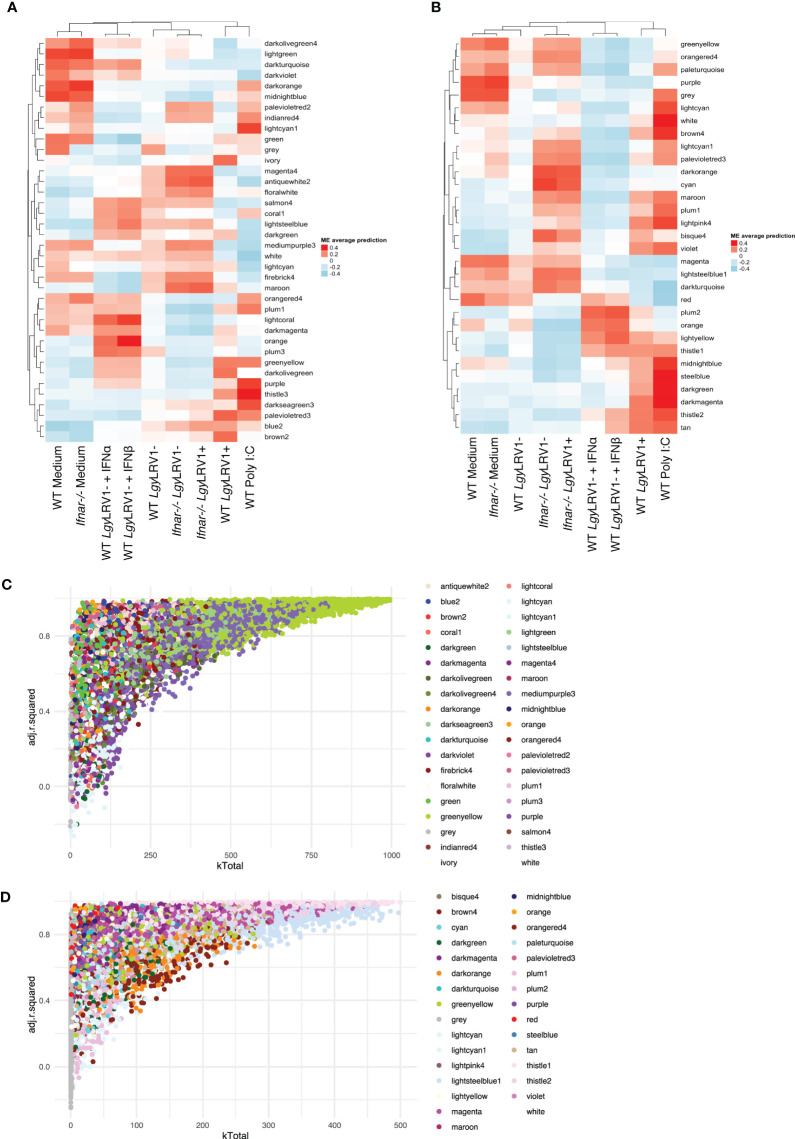
Type I IFNs play a preponderant and central role in the infection mounted by macrophages toward *Lgy*LRV1+. **(A)** Heatmap of the average predictions of the fitted linear model on each ME at the 8-h time point in WT + *Ifnar^-/-^* analysis. **(B)** Heatmap of average predictions of the fitted linear model on each ME at the 24-h time point in WT + *Ifnar^-/-^* analysis. **(C)** Scatter plot of kTotal (whole network connectivity) against adjusted R-squared for all genes in an 8-h network in WT + *Ifnar^-/-^* analysis. Genes are colored according to the module they belong to. **(D)** Scatter plot of kTotal (whole network connectivity) against adjusted R-squared for all genes in a 24-h network in WT + *Ifnar^-/-^* analysis. Genes are colored according to the module they belong to.

Given the importance of the type I IFN response to the immunophenotype observed upon infection with *Lgy*LRV1+ (Rossi et al., 2017) coupled with the observation that modules containing genes with the highest kTotal and adjusted R-squared (8h_bisque4 and 24h_turquoise modules) are enriched in the cellular response to IFN-β (**Figures 3A, B**, **Table 3**) led us to further explore the systemic role of type I IFNs for gene modulation. To achieve this, a WGCNA approach was conducted in the *Ifnar* knockout macrophages (*Ifnar^-/-^)*. Moreover, to directly test the role of type I IFNs, two additional treatments, IFN-α and IFN-β, coadministered after 6 h post- *Lgy*LRV1-infection, were tested to specifically mimic the type I IFN response induced by the *Leishmania* virus LRV1 on the modulation of genes. The analysis generated 38 modules at 8 h p.i. and 31 modules at 24 h p.i., comprising a total of 11,442 and 11,426 genes, respectively (**Figures 4A, B**, *p*-values shown in **Supplementary Figures S6C, D**). As expected, the dendrogram displayed on top of each heatmap showed that modules representing the infection of *Ifnar^-/-^* cells with *Lgy*LRV1+ clustered closer to the modules of WT cells infected with *Lgy*LRV1-, than to the modules of WT cells infected with *Lgy*LRV1+, confirming the major impact that the recognition of type I IFNs had on the macrophages infected by *Lgy*LRV1+. At 8 h p.i. (**Figure 4A**), the addition of type I IFNs to *Lgy*LRV1- did not lead to a drastic effect as these groups clustered closer together with non-treated cells than with *Lgy*LRV1+. This result was not surprising as treatment with type I IFNs was only performed 6 h p.i. with *Lgy*LRV1- and thus cells were only exposed to type I IFNs for 2 h before sample collection. Nevertheless, four modules where the addition of type I IFNs showed an effect by shifting *Lgy*LRV1- closer together to *Lgy*LRV1+ and to poly I:C were identified: 8h_greenyellow (2,226 genes), 8h_mediumpurple3 (1,870 genes), 8h_firebrick4 (479 genes), and 8h_purple (424 genes). These modules contained genes whose expression was promptly modulated by type I IFNs, either exogenously added or produced in response to *Lgy*LRV1+. The heatmap for 24 h (**Figure 4B**) also showed the role of IFNAR in gene modulation as modules representing *Ifnar^-/-^* cells infected with *Lgy*LRV1+ clustered closer to modules representing WT infected with *Lgy*LRV1- rather than *Lgy*LRV1+. Furthermore, at 24 h, cells infected with *Lgy*LRV1- cluster together with non-treated cells. The addition of type I IFNs to *Lgy*LRV1- at 24 h leads to a new cluster further away from non-treated cells contrary to what was observed at 8 h, demonstrating the long-term effect of the type I IFN response. Modules in which the effect of type I IFNs was clearly observed were 24h_tan (579 genes), 24h_thistle2 (744 genes), 24h_thistle1 (680 genes), 24h_lightyellow (266 genes), 24h_darkturquoise (166 genes), 24h_lightsteelblue1 (1,020 genes), and 24h_magenta (1207 genes). Based on the total number of genes detected at 8 and 24 h (11,442 and 11,426, respectively), the percentages of genes modulated by type I IFNs at 8 and 24 h were 43.7% and 40.8%, respectively, showing the drastic impact that type I IFNs have on the concerted modulation of genes.

Following the approach described above, to determine and narrow down the importance of modules, the kTotal (the connectivity of the gene in the whole network) and adjusted R-squared (the proportion of the variance for expression that is explained by infection groups) of each gene were calculated at the two time points and plotted on the x-axis and y-axis, respectively (**Figures 4C, D**). From this analysis, two preponderant modules could explain the variance of the data at 8 h p.i. (modules with the highest kTotal and the highest adjusted R-squared, localized at the tip of the graph), namely, 8h_greenyellow and 8h_mediumpurple3 (**Figure 4C**), and four modules were found at the tip of the graph at 24 h p.i., namely, 24h_thistle1, 24h_thistle2, 24h_lightsteelblue1, and 24h_magenta (**Figure 4D**). Out of these six modules, three were enriched in pathways connected to the immune response, namely, 8h_greenyellow, 24h_thistle1, and 24h_thistle2. These modules were enriched in the following pathways: defense response to virus and protozoan and response to IFN-gamma, -beta, and -alpha; interleukin-6; and tumor necrosis factor productions, whereas the three other ones were enriched in RNA transcription and histone acetylation pathways, as well as in oxidation–reduction and metabolic processes (**Supplementary Table S3**). Curiously, one of the modules identified, the 8h_greenyellow module, was constituted of 2,224 genes, approximately one-fifth of all genes detected at 8 h in the RNA-Seq conducted, showing not only the importance but also the coordination of the early immune response in macrophages upon a challenge. Given the influence of hub genes in orchestrating the response of the modules, the top 1% genes with the highest kWithin of these two and four modules were examined at 8 and 24 h p.i. (**Table 4**). Interestingly, as examples *Cxcl11*, *Tgtp1*, and *Il27* (in the greenyellow module) at the 8-h time point; *Ifih1* and *Igtp* (in the thistle1 module), *Il15ra* and *Jak2* (in the thistle2 module) at the 24-h time point were found (**Table 4**) (for the whole lists of genes with their module membership and their connectivity at each time point, see **Supplementary Table S1**).

**Table 4:** The top 1% genes with the highest kWithin of the preponderant modules that explain the variance of the data in WT + *Ifnar^-/-^* analysis at 8 and 24 h p.i.

Time point	Module	Top 1% genes
8h	greenyellow	*Tbc1d13, Mier3, Cxcl11, Tgtp1, Papd4, Snx2, Zfp319, Prpf38a, Mpp1, Hdc, Plekha2, Fbxw11, G3bp2, March5, Dync1i2, Il27, Khdrbs1, Mxd1*, *Fbxo7, Rnf31, A630012P03Rik, Gnb4, Snw1*
	mediumpurple3	*Mlec, Arrb1, Fbxo31, Scd2, Trim37, Lactb2, Ddhd2, Zfp146, Klc4, Mcm2, Rrp1b, 2410002F23Rik, Polrmt, Trmt2a, Mettl13, Nfx1, Lpin1, Rxra, Aco1*
24h	thistle1	*Ifih1, Sp110, Daxx, Samhd1, Tor1aip1, Igtp, Parp12*
	thistle2	*Rnf19b, Il15ra, Tbc1d13, Ttc9c, Peli1, Rapgef2, Jak2, Cflar*
	lightsteelblue1	*Slc25a4, Amz1, Rpl3, Zfp664, Mgst3, Gusb, Pabpc4, Smyd2, Sfxn1, Fgfr1, Mlec*
	magenta	*Ank, Aldh9a1, Adcy9, St6gal1, Man1c1, Igfbp4, Zfyve28, Klhl42, Sh3pxd2a, Prkar2a, Pygl, Deptor, B3glct*

### Overlap of highly connected modules at early and late time points uncovered the temporal dynamics of the interferon response

**Figure 5:**
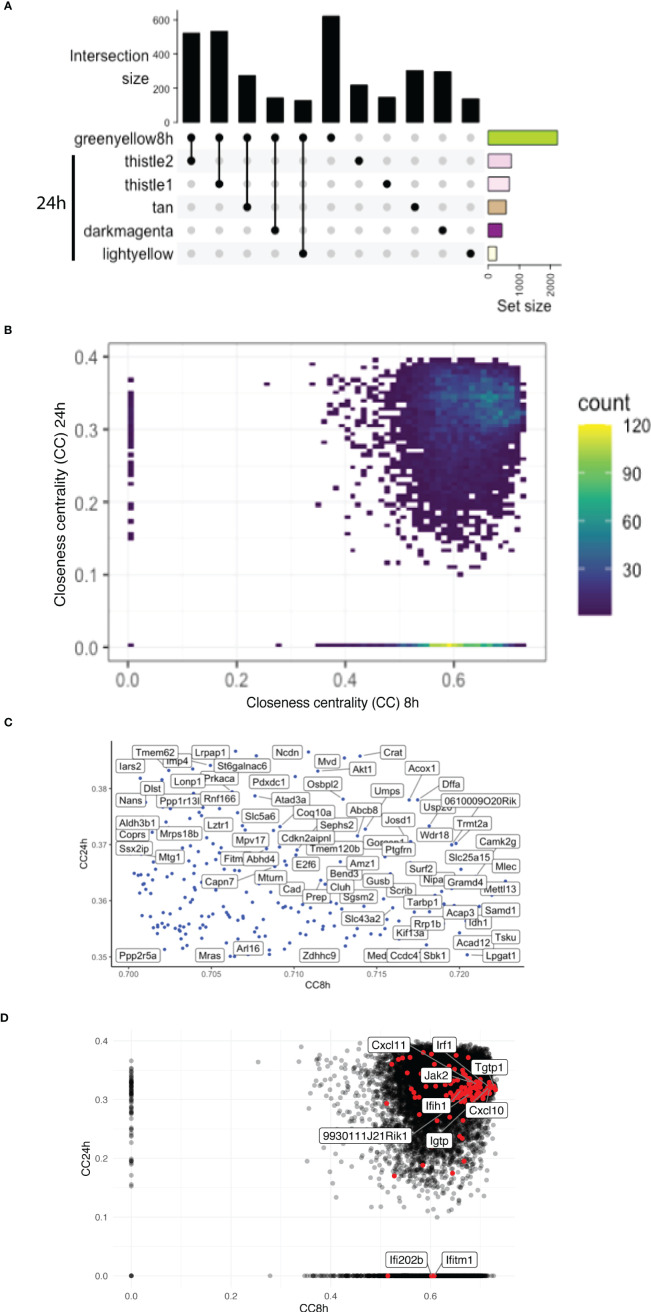
Overlap of highly connected modules at early and late time points uncovers the temporal dynamics of the interferon response. **(A)** An UpSet plot of the 8h_greenyellow module overlapping with the modules at the 24-h time point in WT + Ifnar-/- analysis. Top five overlapping modules are shown. Intersection size is shown in the y-axis. The bottom-right part shows the total module size. **(B)** Density plot of the CC of genes in the WGCNA network at 8 h (x-axis) against 24 h (y-axis) in WT + Ifnar-/- analysis. Count unit corresponds to the number of genes in each rectangle. **(C)** Zoom of the tip of the CC plot. Genes with very high centrality at both 8 and 24 h p.i. **(D)** Scatter plot of CC of genes in the WGCNA network at 8 h (x-axis) against 24 h (y-axis) in WT + Ifnar-/- analysis. Positions of ISGs are highlighted in red and the names of 10 examples are shown.

The greenyellow module identified at 8 h that represented a large proportion of the genes identified is also enriched in different Gos, including many related to immunity. However, at 24 h p.i., more than one module was enriched in Gos connected to immunity (**Supplementary Table S3**). To understand the temporal dynamics of the macrophage response, the overlap between the major module identified at 8 h and the modules identified at 24 h p.i. in the WT + *Ifnar^-/-^* analysis was evaluated. Interestingly, the upset graph (**Figure 5A**) showed that the 8h_greenyellow module overlapped very strongly with 24h_thistle1 and 24h_thistle2, 24h_tan, 24h_darkmagenta, and 24h_lightyellow. As previously mentioned, the striking GO terms enriched in immune-related Gos were found in 8h_greenyellow, 24h_thistle1, and 24h_thistle2. The predominant terms identified in the 8h_greenyellow module were also found at 24-h split into the 24h_thistle1 module (e.g., defense response to virus and protozoan, innate immune response, and cellular response to IFN-gamma and -beta) and 24h_thistle2 (e.g., a positive regulation of the ERK1 and ERK2 cascades, interleukin-12, and chemokine productions) (**Supplementary Table S3**). Intriguingly, other Gos that demonstrated a concerted modulation in 8h_greenyellow and were thus associated with initial immune responses were now split into 24h_tan (e.g., a negative regulation of the NF-kappaB transcription factor activity), 24h_darkmagenta (e.g., a positive regulation of the inflammatory response) and 24h_lightyellow (e.g., the regulation of cytokine secretion and the MyD88-dependent Toll-like receptor signaling pathway) (**Supplementary Table S3** and data not shown). This latter part was interesting in terms of Gos that can be initially associated with the immune response toward invaders but were then separated. Remarkably, some other GO terms of the 8h_greenyellow module were not found significantly (*p*-value < 0.01) in any of the five modules cited above and identified in **Figure 5A** (e.g., a negative regulation of interleukin-10 production). These results pointed toward the dynamic nature of the response, being more concerted at 8 h and then split into the groups of genes with slightly different modulations and thus ending up in different modules.

**Figure 6:**
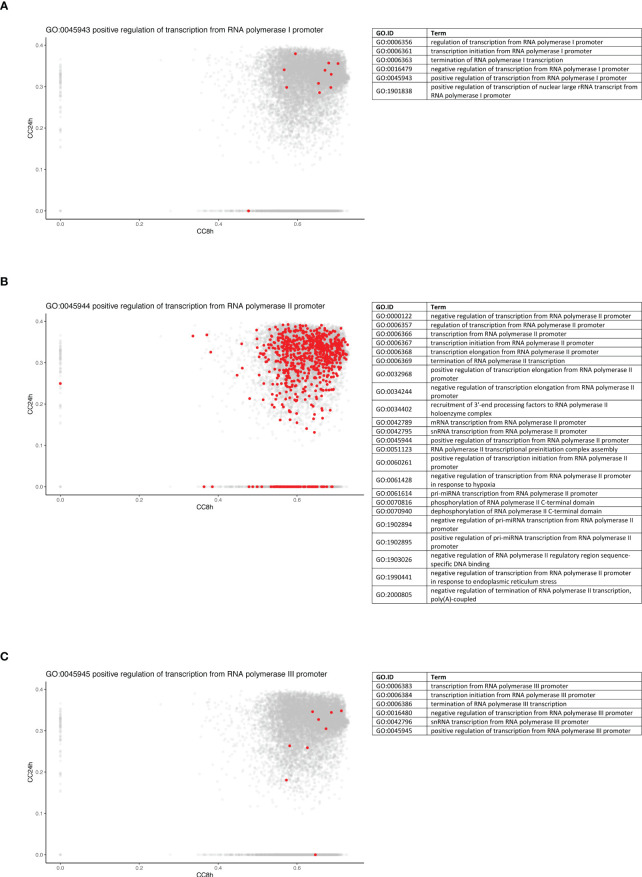
Genes from RNA polymerase II pathway are predominantly central at early time point. Density plots of the CC of genes in the WGCNA network at 8 h (x-axis) against 24 h (y-axis) in WT + *Ifnar^-/-^* analysis. Genes belonging to the examples of RNA polymerase I **(A)**, II **(B)**, and III **(C)** processes are highlighted in red. The lists of GO terms found for RNA polymerase I (Table A), II (Table B), and III (Table C) keywords are listed.

To further dissect the temporal dynamic of the macrophage response toward infection, the CC of each gene in the network was calculated at 8 and 24 h p.i., then plotted on x- and y-axes, respectively. This measure allowed us to identify genes that were best placed to influence the entire network as regulators and important signal transducers. The different categories of genes regarding their position on the CC plot are shown in **Figure 5B**. Strikingly, there were genes with very low centrality (below 0.05) at 8 h (on the y-axis) and at 24 h (on the x-axis), 76 genes versus 1567 genes, respectively (approximately 20 times more), suggesting that these genes were essential at 24 h versus 8 h p.i., respectively. The great difference in the number of genes in these two groups showed that many central events could happen at 8 h, right at the beginning of the infection, losing its importance later on or setting the stage for the later events. At 8 h, the predominant GO terms were related to the RNA polymerase II pathway such as the positive regulation of transcription and transcription initiation from the RNA polymerase II promoter (**Figure 6**). To sum up, 23 terms were enriched in RNA polymerase II processes in comparison to only 12 terms related to both RNA polymerase I and III pathways (for example, a positive regulation of the transcription from the RNA polymerase I promoter and RNA polymerase III promoter, respectively) (**Figure 6**). In contrast, no relevant GO term was found enriched in the set of 76 genes with high CC at 24 h p.i. and very low at 8 h p.i. On the CC plot, the blue-to-yellow scale (density) represents the number of genes per position. A “hotspot” with the highest density, localized on the x-axis (i.e., with very low CC at a 24-h time point), with a CC value approximately 0.6 at an 8-h time point was pinpointed. This “hotspot” of genes belonged to the 1,567 genes mentioned before (with very low centrality at 24 h p.i.).

The tip of the CC plot was defined by genes with very high centrality both at 8 and at 24 h p.i. The genes present at the tip of the CC plot belonged to the modules 8h_greenyellow, 8h_lightgreen, and most importantly 8h_mediumpurple3 (**Figure 5C**). As mentioned previously, this last module was globally enriched in GO terms linked to transcription, meaning that these pathways initially influenced the network the most. While there were three modules from the 8-h modules, the genes at the tip were divided into more than 10 modules from the 24-h modules (higher than four times more). For example, the tip of the CC plot included Akt1, a member of the protein kinase B family, known to be involved in macrophage survival and parasite persistence (Eren et al., 2016).

Given the high and central importance of type I IFNs in the modulation of the transcript response and the outcome of an infection by LgyLRV1+, a non-exhaustive list of genes stimulated by type I IFNs was established from the GO database and some other references and their position on the CC plot evaluated (**Figure 5D**). Genes from three GO terms were compiled: cellular response to IFN-alpha (GO:0035457), cellular response to IFN-beta (GO:0035458), and cellular response to type I IFN (GO:0071357). In addition, genes cited in different publications were also considered (Zhao et al., 2009; Schoggins and Rice, 2011; Rand et al., 2012; Cheon et al., 2014; Manjunath et al., 2017; Nasr et al., 2017; Pervolaraki et al., 2018; Ashley et al., 2019; Yang et al., 2020). Thus, the list comprised a total of 175 genes analyzed at 8 and 24 h p.i. in WT + *Ifnar^-/-^* analysis. Among them, 139 and 138 genes were detected at 8 and 24 h p.i., respectively. The consistency of detection between early and late time points showed a sustained induction of IFN-stimulated genes (ISGs). However, there was one gene detected at 8 h p.i. but not at 24 h p.i.: *Ifi202b* (**Supplementary Table S4**). The membership status of the whole list of detected genes was then investigated, and, as expected, the main modules related to immune pathways were predominant. More than half of the ISGs detected at 8 and 24 h p.i. belong to the 8h_greenyellow (96 genes) and its overlapping 24h_thistle1 and 24h_thistle2 modules (70 + 20 = 90 genes), respectively. Nevertheless, the early gene, *Ifi202b*, belonged to the 8h_blue2 module (**Supplementary Table S4**). To confront these ISGs to the CC analysis, the 175 genes were sought, and the detected ones are highlighted (in red in **Figure 5D**). The five top genes were *Myd88*, *Tgtp1*, *Mov10*, *Trim21*, and *Samhd1* showing the highest CC values at 8 h p.i. (>0.72) and *Irak1*, *Sun2*, *Ttll12*, *Mmp12*, and *Ube2g2* with the highest CC values at 24 h p.i. (>0.37) (**Figure 5D**, **Supplementary Table S4**). *Ifi202b* has a CC value of 0.60 at 8 h p.i. and is localized on the x-axis; thus, it is part of the “hotspot” already defined before (**Figure 5B**). Even if detected at 24 h p.i., another ISG with a very low CC value (below 0.05) at 24 h p.i. belonged to this “hotspot” and was found on the x-axis: *Ifitm1* (8h_CC value of 0.61) (**Figures 5B, D**, **Supplementary Table S4**). *Ifitm1* was part of the 8h_plum1 module, while, as expected at 24 h p.i., it was included in 24h_grey, a label reserved to genes that was not part of any module (**Supplementary Table S4**). Therefore, these two genes, *Ifi202b* and *Ifitm1*, seemed to be very central at the early time point, suggesting that they were both important at 8 h p.i. and not at 24 h p.i.

## Discussion

Many studies have addressed, using RNA-Seq, the transcriptional changes in the macrophage response upon *Leishmania* infection (Salloum et al., 2021). Traditionally, these studies have investigated differentially expressed genes with significantly altered expression across the groups of samples. However, moving from a gene-by-gene analysis to function is very challenging. In that regard, network analysis methods, such as WGCNA, have the advantage to provide a comprehensive view at the system level. As an unsupervised algorithm, WCGNA can establish and detect the relationship between gene expression and phenotypic traits. Moreover, WGCNA has the advantage over other methods in analyzing multivariate and complex datasets (Langfelder and Horvath, 2008). Indeed, WGCNA allows to condense a large network of genes into a limited number of variables (i.e., modules). Furthermore, WGCNA permits the identification of hub genes that play a central role in driving the system of interest. While an WGCNA approach has already been conducted in the blood of patients (Gardinassi et al., 2016) and the popliteal lymph nodes of dogs (Sanz et al., 2021) infected with *L. infantum* as well as in the cutaneous lesions of *L. braziliensis–*infected patients (Christensen et al., 2016) and *L. major–*infected human dendritic cells (Zhao et al., 2019), no WGCNA investigation has been piloted on *Leishmania*-infected macrophages. Aside from being, to our knowledge, the first study where WGCNA was conducted in *Leishmania*-infected macrophages *in vitro*, this study also represents the first comprehensive transcriptional study addressing the response mounted by macrophages upon infection by *Lgy*LRV1+, thus addressing the impact the presence of LRV1 has on the macrophage response (Cantacessi et al., 2015; Salloum et al., 2021). The present WGCNA identified biologically relevant groups of transcripts, classified into modules, that were modified upon macrophage infection by *Lgy*LRV1- and *Lgy*LRV1+ after 8 or 24 h of infection. These modules were further analyzed and key pathways and hub genes associated with *Leishmania*, with LRV1 or with the exacerbatory effect of LRV1 in *Lgy*LRV1+, were identified. Additionally, the impact of type I IFNs on the transcriptional profile of macrophages upon *Lgy*LRV1+ infection was evaluated.

### The major impact of LRV1 and the relevance of type I interferons

Globally, this study showed that the presence of LRV1 had a major impact in the transcriptomic profile of macrophages both at 8 and 24 h p.i. The LRV1 and the “exacerbatory” modules, that is, modules reflecting the effect of the presence of LRV1 on top of the presence of *Leishmania*, were identified and shown to represent a very considerable part of the overall transcriptomic change (representing together 53.3% and 42.5% at 8 and 24 h p.i.). The immunophenotype associated to the presence of LRV1, or to other viruses, was shown to be highly dependent on the systemic production of type I IFNs (Rossi et al., 2017; Rossi and Fasel, 2018b; Rath et al., 2019; Heirwegh et al., 2021). Although type I IFNs, such as IFN-α and IFN-β, are crucial for viral clearance, their influence during a *Leishmania* infection appears to be highly contextual. Type I IFNs have a worsening effect on the outcome of *L. guyanensis*, while in the case of *L. major*, the addition of type I IFNs promotes the resolution of infection (Hartley et al., 2014). Here, we show the massive role that the production of type I IFNs downstream of the recognition of LRV1 has at a transcriptional level influencing 43.7% and 40.8% of all transcriptional changes at 8 and 24 h p.i. Type I IFNs are produced by different cell types including macrophages and can act both in an autocrine and paracrine manner. The recognition of type I IFN by the IFNAR receptor (a complex composed of two subunits: IFNAR1 and IFNAR2) initiates a signaling cascade, leading to the expression of a vast panel of ISGs (IFN-stimulated genes) with a positive feedback loop and also direct antiviral effects (Sadler and Williams, 2008). Many studies have investigated the induction of some ISGs in response to a viral infection, for example, adenovirus, morbillivirus, retrovirus, vesiculovirus, herpesvirus, and paramyxovirus infections (Zhao et al., 2009; Manjunath et al., 2017; Nasr et al., 2017; Pervolaraki et al., 2018; Ashley et al., 2019; Yang et al., 2020). Interestingly, some of the hub genes found in the WT and WT + *Ifnar^-/-^* analyses (**Figure 2**, **Table 4**) were part of the ISGs such as *9930111J21Rik1*, *Cxcl10*, *Cxcl11*, *Ifih1*, *Igtp*, *Irf1*, *Jak2*, and *Tgtp1* in WT + *Ifnar^-/-^* analysis (**Supplementary Table S4**). The chemokine CXCL10 has been demonstrated to be linked to the endosymbiotic LRV1 (Ives et al., 2011; Kariyawasam et al., 2017) and the *Leishmania* evasion from the host (Antonia et al., 2019).

### TNF-α and IL-6 regulation as evidence of the crosstalk between modules

Hyperinflammation is a hallmark of an *Lgy*LRV1+ infection (Ives et al., 2011). Two major mediators of inflammation contributing to this immunophenotype are the cytokines IL-6 and TNF-α. Surprisingly, these two molecules were found present in two distinct modules: IL-6 was found in the 8h_coral 3 (an LRV1-associated module), while TNF-α was found associated to the 8h_bisque4 modules (an “exacerbatory” module of WT analysis). In addition to TNF-α, CXCL10 (one of the top five hub genes), and CCL5, two chemokines also found to be the hallmarks of the inflammation caused by *Lgy*LRV1+ infection were also found to be present in the 8h_bisque4. Interestingly, even though IL-6 is not found in the 8h_bisque4, this module was shown to be enriched in the GO term “positive regulation of IL-6.” Further analysis of both 8h_bisque4 and 8h_coral3 modules revealed that the members of the Src-family kinase (SFK) were also found in these two modules. Src and Hck were also found present in the 8h_bisque 4 together with TNF-α, whereas the other members of the SFK-family such as Lyn and Lck were found present in the 8h_coral3 module, the module that contains IL-6. This specific distribution of SFK is particularly relevant since, from our previous studies, it was shown that KB-SRC4, a specific inhibitor of c-Src, blocks IL-6 but not TNF-α (Brandvold et al., 2012; Reverte et al., 2021). On the contrary, PP2, which efficiently blocks Fyn, Hck, and Lck, inhibits TNF-α but not IL-6 secretion (Hanke et al., 1996; Reverte et al., 2021). Thus, it is interesting to underline not only the relevance of SFKs in TNF-α and IL-6 secretion but also the crosstalk between two modules containing genes upregulated in *Lgy*LRV1+-infected cells. Interestingly, Csk, the kinase that regulates the intramolecular inhibition of SFKs, was also identified in the 8h_coral3 module. This module also includes important genes, such as IL-1 or IFN-γ previously associated with the presence of LRV1 (Ives et al., 2011; Hartley et al., 2016; Rossi et al., 2017; Kariyawasam et al., 2017; Castiglioni et al., 2017) as well as other genes *Il18*, *Ccl8*, *Socs 2*, *Casp3*, *Il10*, *Jun*, *Jak3*, *Bcl10*, and *Ly6e* implicated in the response to *Leishmania* infection. Overall, these data highlight a physiological and phenotypically relevant crosstalk between two different modules. Moreover, since the role of IL-6 has been considered, by Osero et al., one of the greatest oxymora in the leishmaniasis outcome due to different studies showing discordant roles (Osero et al., 2020), this crosstalk also pointed toward the relevance that a module associated in this study to an LRV1 module may contribute to the leishmaniasis outcome.

### IL-15 as a possible important hub

As mentioned above, the WGCNA analysis identifies hub genes that play a central role in a given module, that is, genes most likely to influence the whole module and thus drive its enriched pathways. Among the hubs identified, one gene, found in both analyses, is *Il15ra*, encoding for the receptor of the interleukin, IL-15 (**Figure 2**, **Table 4**). This cytokine, in combination with IL-12, stimulates the cellular immune response in dogs and humans with visceral leishmaniasis (Milano et al., 2002; Costa et al., 2020). Similarly, another cytokine-related gene was observed as a driver gene but only in WT + *Ifnar^-/-^*, for example, *Il27*. IL-27 is associated with resistance or susceptibility to *Leishmania* infection (Jafarzadeh et al., 2020) and to the antiviral response to several RNA viruses, such as HIV, Hepatitis C Virus (HCV), or Chikungunya Virus (CHIKV) (Valdés-López et al., 2022). Interestingly, *Il15*, *Il15ra*, *Il12a*, *Il12b*, and *Il27* genes were all present in the same “exacerbatory” module at 8 h p.i. (8h_bisque4), then separated into the 24h_brown (for *Il15* and *Il15ra*) and 24h_turquoise (for *Il12b* and *Il27*) modules, associated to LRV1 and exacerbatory phenotypes in WT analysis.

### Activation of specific transcription factors in response to LRV1

Given the described importance of LRV1 in the leishmaniasis outcome, progression, relapse, and treatment failure, hub genes associated to the presence of LRV1 are of utmost interest when designing strategies to fight leishmaniasis (Hartley et al., 2014; Adaui et al., 2016; Bourreau et al., 2016; Rossi et al., 2017). Several relevant transducers and transcription factors were present in the 8h_coral3 and 8h_bisque4 modules in WT analysis (e.g., Myd88, Keap1, Tank, Ikbke, Stat1, Stat2, Irf1, Irf5, Irf7, Ets2, and Xbp1). For example, *Irf1* was part of the “exacerbatory” 8h_bisque4 module. IRF1 participates in the expression of cytokines such as CCL5 and CXCL10. The lack of IRF1 shows a dramatically exacerbated leishmaniasis disease in *L. major–*infected mice associated with a decrease of IFN-γ and IL-12 productions (Lohoff et al., 1997). IRF1 is also involved in antiviral responses against dsRNA viruses, for example, HCV (Pflugheber et al., 2002; Feng et al., 2017). Furthermore, *Igf1r* was a driver gene in WT analysis and belonged to the 24h_yellow module and the “exacerbatory” group. Similarly, the insulin-like growth factor 1 receptor (IGF1R) has previously been shown to be involved in arginase (Arg1) expression in visceral leishmaniasis (Osorio et al., 2014) and also targeted by rotaviruses, dsRNA viruses, to manipulate the PI3K/Akt pathway and block autophagy (Zhou et al., 2018). More intriguingly, several transcription factors were found among the list of these hub genes in WT analysis, such as *Sp1*. Sp1 that belonged to the 24h_plum1 is involved in the evasion mechanisms of *Leishmania*. Different species can induce the binding of Sp1 on *Il10*, *Ucp2*, and *Hdac1* promoters in macrophages, resulting in the production of the inhibitory IL-10 cytokine, the suppression of oxidative burst, and a decrease of iNOS expression, respectively (Yang et al., 2007; Basu Ball et al., 2014; Calegari-Silva et al., 2018).

### Interferon-stimulated genes and temporal expression

Some studies have analyzed different time points following an infection challenge and revealed that IFN-stimulated genes (ISGs) can be temporally expressed: earlier or later, in order to specifically target and counteract different steps of the virus life cycle (Zhao et al., 2009; Manjunath et al., 2017; Nasr et al., 2017; Pervolaraki et al., 2018). The analysis of the closeness centrality of some ISGs brought two genes to the forefront, *Ifi202b* and *Ifitm1*, which were shown to be central at an 8-h time point only (**Figure 5**, **Supplementary Table S4**). Therefore, these two candidates could be decisive in the early events happening during the cellular response and could represent new targets to further investigate. On one hand, *Ifi202b*, also known as IFN-activated gene 202B and encoding for the P202 protein, belongs to the pyrin and HIN domain–containing (PYHIN) proteins, as AIM2 and IFI16 (Wang et al., 2018). In several studies, *Ifi202b* has been associated with sex differences in autoimmune diseases (Panchanathan et al., 2011; Cao et al., 2018) and this question has recently been addressed in the *Leishmania* inflammation context (Lockard et al., 2019; Snaka et al., 2022). Likewise, *Ifi202b* has been shown to be upregulated upon *L. major* footpad infection in C57BL/6 mice (Ehrchen et al., 2010) and its corresponding P202 protein to exert an inhibitory effect on the AIM2 inflammasome (Wang et al., 2018). On the other hand, Ifitm1, encoding the IFN-induced transmembrane protein 1 (IFITM1), is known to have a role in the restriction of many RNA viruses, such as HIV (Chutiwitoonchai et al., 2013) or HCV (Narayana et al., 2015).

### Toward strategies to face *Leishmania*-virus coinfections and beyond

The apparent disproportional response toward a non-pathogenic element such as LRV1 has the potential to change the outcome of the infection by a pathogenic element such as *Leishmania.* Although LRV1 is only present in some strains of *Leishmania*, the results presented here can have a broader impact: the modulation of leishmaniasis by endogenous or exogenous viruses is particularly relevant as the vector of *Leishmania*, the phlebotomine sand flies, also carries phleboviruses such as Toscana virus shown previously to cause the same phenotype as the presence of LRV1 when coadministered with *Leishmania*. Additionally, the effect that virus can have go beyond LRV1 or the coadministration of phlebovirus during a sand fly bite. Mice challenged with a virus, such as LCMV, upon the resolution of *Leishmania* infection, led to a relapse of the *Leishmania* infection showing that exacerbation and the metastatic phenotype are not always linked to the presence of LRV1 (Valencia et al., 2022) but could also be related to the presence of other viruses (Rossi et al., 2017; Rath et al., 2019; Heirwegh et al., 2021). By exposing the modules and pathways affected by LRV1 as well as the hub genes responsible for driving such events, this study can contribute to the future design of strategies to deal with a *Leishmania*-viral coinfection.

The RNA-Seq data used in this manuscript are available to all communities through the website https://amelbek.shinyapps.io/fasel_lab_data/ where individual or groups of genes can be visualized in different genotypes of macrophages (as the data for this study were part of larger datasets containing additional mouse genotypes IFNAR-/-, IFNg-/-, iNOS-/-, NLRX1-/-, NOX2-/-, and PRX5-/-). The data can be downloaded for further statistical analysis. Thus, this study and these datasets also constitute a resource that can be further explored to study the impact of *Leishmania* on the macrophage response and on the importance of viral coinfections or type I IFNs and beyond in the outcome of leishmaniasis.

## Data availability statement

The datasets presented in this study can be found in online repositories. The names of the repository/repositories and accession number(s) can be found below: https://www.ncbi.nlm.nih.gov/geo/, GSE201120, https://www.ncbi.nlm.nih.gov/geo/, GSE203088.

## Ethics statement

The animal study was reviewed and approved by Veterinary Commission of the Canton de Vaud (SCAV, Switzerland).

## Author contributions

NI, FT, TS, CD, DK, FP, and MR performed experiments. AB analyzed the data. FT, AB, and NI wrote the manuscript. FT, AB, NI, IX, and NF interpreted and discussed the data. TS, SC, IX, and NF reviewed and edited the manuscript. All authors contributed to the article and approved the submitted version.

## Funding

This work was supported by the grant from the Swiss National fund for research to NF (Grant No. 310030_173180) and the grant from Foundation Pierre Mercier pour la science to FT.

## Acknowledgments

The authors thank the veterinary and animal facility staff at CIIL for ensuring animal welfare and ethical standards. Library preparation, sequencing and raw data normalization were performed at the Lausanne Genomic Technologies Facility (GTF), University of Lausanne (UNIL), Switzerland (https://wp.unil.ch/gtf/). The authors specially thank the following members of the facility for their support: Leonore Wigger, Sandra Calderon, Julien Marquis, Hannes Richter, Johann Weber, Roberto Sermier, and also the former members, Sylvain Pradervand, Floriane Consales Barras and Keith Harshman. The authors thank Matteo Rossi for his help with the initiation of the study and Alexander Miesch for his precious assistance and patience in making the data available. The authors are very thankful to Slavica Masina for her critical reading.

## Conflict of interest

The authors declare that the research was conducted in the absence of any commercial or financial relationships that could be construed as a potential conflict of interest.

## Publisher’s note

All claims expressed in this article are solely those of the authors and do not necessarily represent those of their affiliated organizations, or those of the publisher, the editors and the reviewers. Any product that may be evaluated in this article, or claim that may be made by its manufacturer, is not guaranteed or endorsed by the publisher.
