# Supplementary material for: Dissection of the macrophage response towards infection by the Leishmania-viral endosymbiont duo and dynamics of the type I interferon response
Source: Front Cell Infect Microbiol. 2022 Aug 4;12:941888. doi: 10.3389/fcimb.2022.941888 (PMC9386148; doi:10.3389/fcimb.2022.941888)

**Figure S1. The distributions of dataset#1 and dataset#2 have similar patterns at 8- and 24-hours post-infection and the different groups of conditions are well separated.** Density plots of the expression values (log cpm). Dataset#1 is shown in red & dataset#2 in blue, both at 8 hours (A) and 24 hours (B) post-infection. Principal component analysis of the conditions showing PC1 and PC2 for dataset#1 (circle) and dataset#2 (triangle) at 8 hours (C) and 24 hours (D) post-infection.


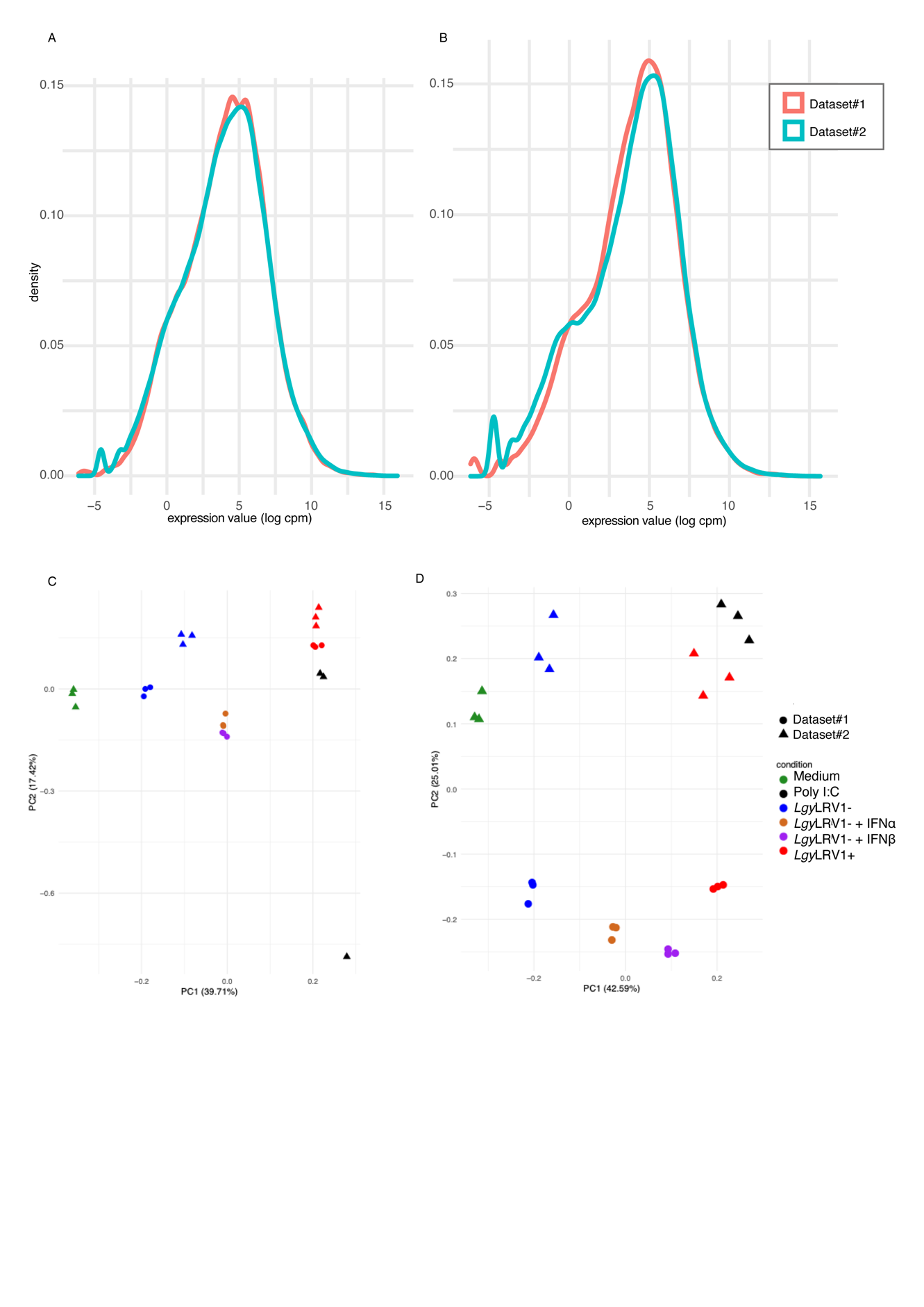

Supplement: Supplementary file 1 [file DataSheet_1.zip › Data Sheet 1/Supplementary Material/Figure S1.docx]
