# Supplementary material for: Dissection of the macrophage response towards infection by the Leishmania-viral endosymbiont duo and dynamics of the type I interferon response
Source: Front Cell Infect Microbiol. 2022 Aug 4;12:941888. doi: 10.3389/fcimb.2022.941888 (PMC9386148; doi:10.3389/fcimb.2022.941888)

**Figure S4.** **Different steps of the WGCNA for the WT + *Ifnar^-/-^* analysis at 8 hours post-infection.** A. Scale of independence plot showing analysis of the scale free topology model fit for various soft-thresholding powers. B. Mean connectivity represented for various soft-thresholding powers. C. Eigengene Dendogram showing 91 dynamic modules yielded 38 main clusters. D. Hierarchical cluster tree of genes in the weighted gene co-expression network. Each short vertical line corresponds to a transcript, and the branches are expression modules of highly interconnected groups of genes, with a color to indicate the module assignment.


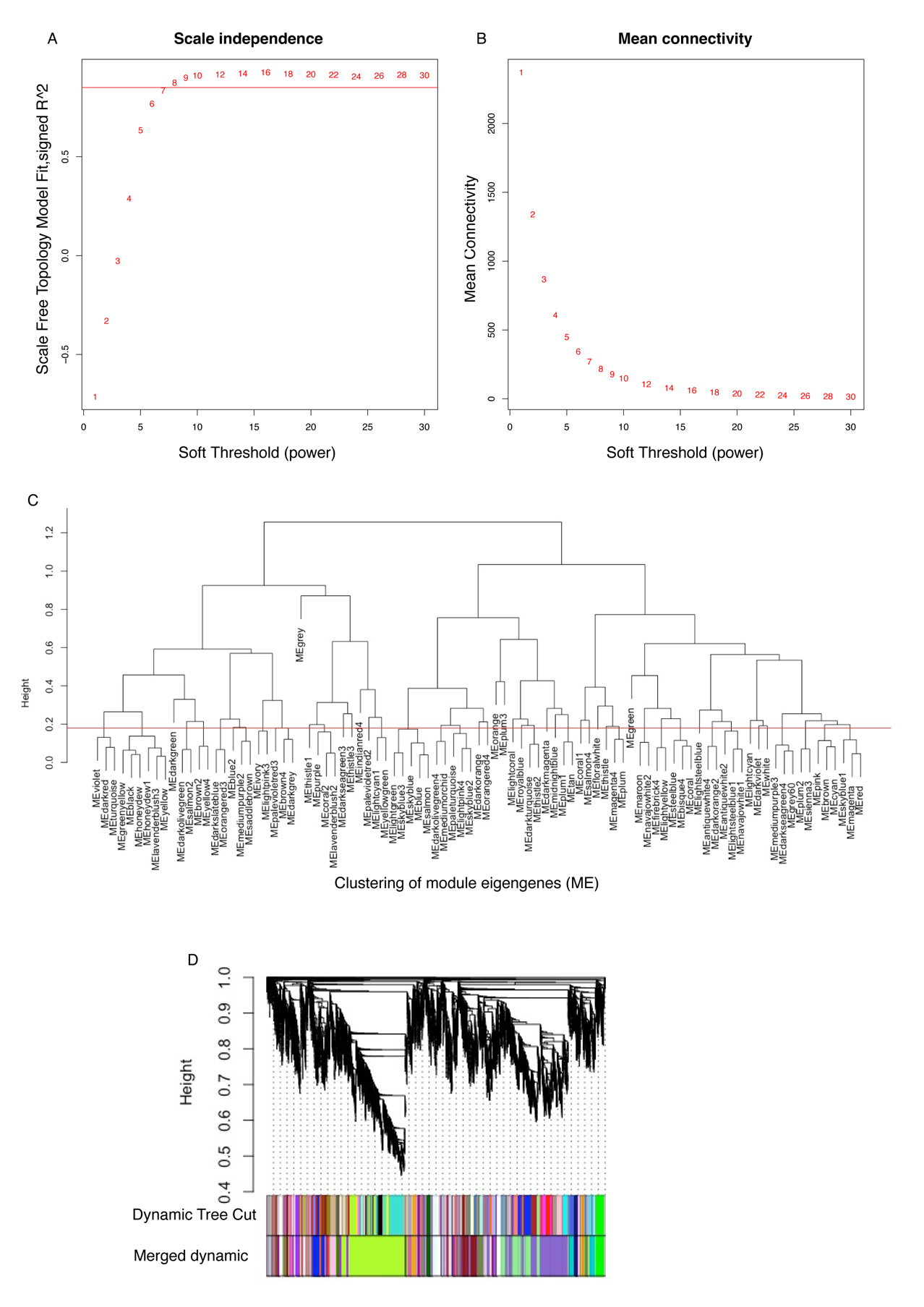

Supplement: Supplementary file 1 [file DataSheet_1.zip › Data Sheet 1/Supplementary Material/Figure S4.docx]
