# Supplementary material for: Dissection of the macrophage response towards infection by the Leishmania-viral endosymbiont duo and dynamics of the type I interferon response
Source: Front Cell Infect Microbiol. 2022 Aug 4;12:941888. doi: 10.3389/fcimb.2022.941888 (PMC9386148; doi:10.3389/fcimb.2022.941888)

**Figure S6.** **Heatmaps showing *p*-values.** Heatmaps of average predictions of fitted linear model on each module eigengenes at 8h time point in WT analysis (A), at 24h time point in WT analysis (B), at 8h time point in WT + *Ifnar^-/-^* analysis (C) and at 24h time point in WT + *Ifnar^-/-^* analysis (D). *p*-values are shown in each rectangle.


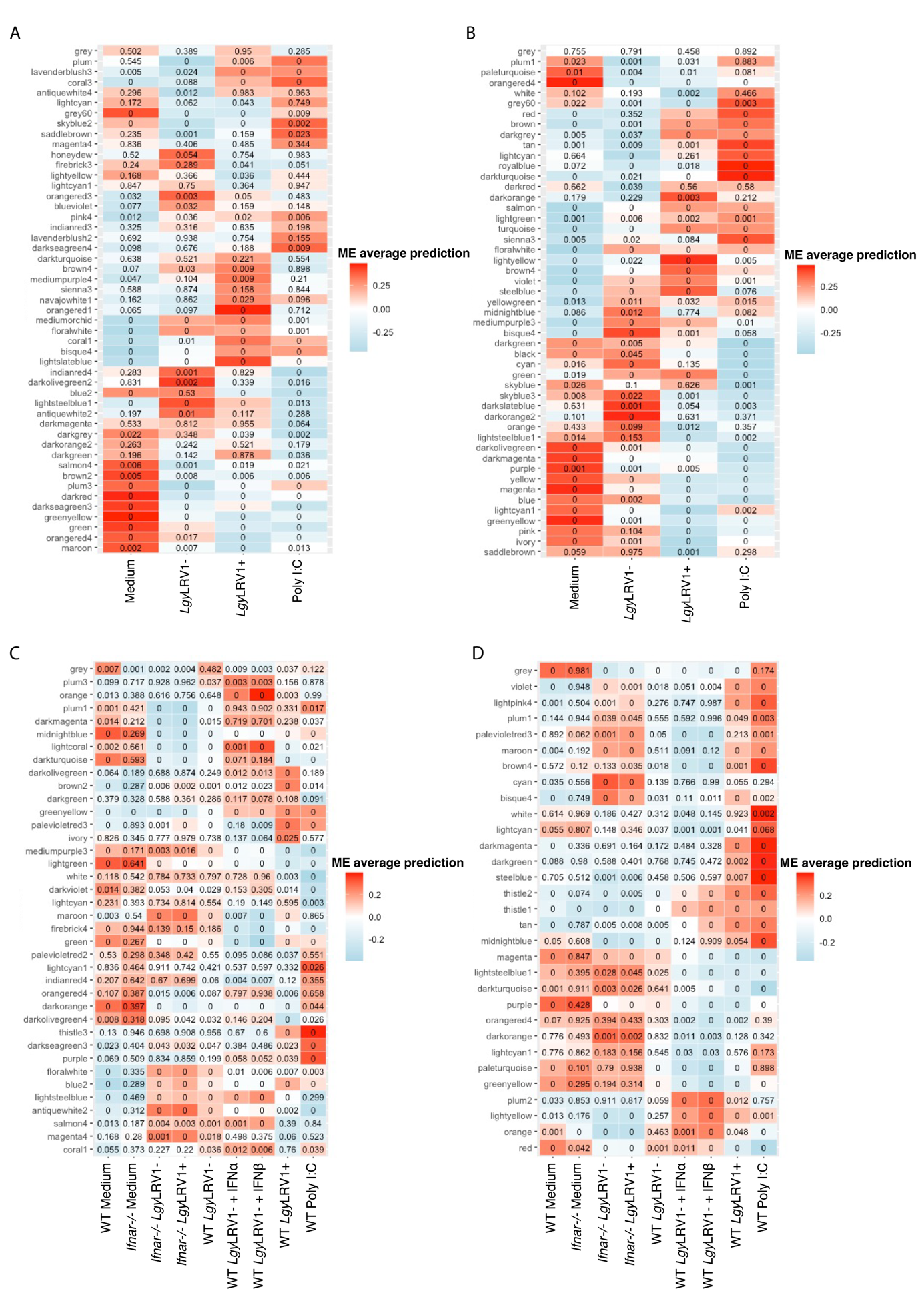

Supplement: Supplementary file 1 [file DataSheet_1.zip › Data Sheet 1/Supplementary Material/Figure S6.docx]
