# Supplementary material for: Dissection of the macrophage response towards infection by the Leishmania-viral endosymbiont duo and dynamics of the type I interferon response
Source: Front Cell Infect Microbiol. 2022 Aug 4;12:941888. doi: 10.3389/fcimb.2022.941888 (PMC9386148; doi:10.3389/fcimb.2022.941888)
Supplement: Supplementary file 1 [file DataSheet_1.zip › Data Sheet 1/Supplementary Material/Table S2.docx]

**Table S2. Examples of main GO terms of modules associated with *Leishmania*, LRV1 or both agents (“exacerbatory”) in WT analysis (biological process “BP” category and *p*-value < 0.01) at 8- and 24-hours post-infection.**

| **Group** | **Time** | **Module** | **GO.ID** | ***p*-value** | **Term** |
| --- | --- | --- | --- | --- | --- |
| *Leishmania* | 8h | grey60 | GO:1904355 | 2.25E-03 | positive regulation of telomere capping |
|  |  |  | GO:0006284 | 6.10E-03 | base-excision repair |
|  |  | floralwhite | GO:0010501 | 3.38E-04 | RNA secondary structure unwinding |
|  |  |  | GO:0046931 | 7.09E-04 | pore complex assembly |
|  |  |  | GO:0045651 | 3.16E-03 | positive regulation of macrophage differentiation |
|  |  |  | GO:0042744 | 4.58E-03 | hydrogen peroxide catabolic process |
|  |  |  | GO:0090281 | 4.58E-03 | negative regulation of calcium ion import |
|  |  | lightcyan | GO:0009215 | 5.86E-05 | purine deoxyribonucleoside triphosphate metabolic process |
|  |  |  | GO:0009151 | 8.33E-05 | purine deoxyribonucleotide metabolic process |
|  |  |  | GO:0034508 | 5.43E-03 | centromere complex assembly |
|  |  |  | GO:0006810 | 8.45E-03 | transport |
|  |  | magenta4 | GO:0006749 | 3.53E-03 | glutathione metabolic process |
|  | 24h | mediumpurple3 | GO:0002092 | 5.31E-04 | positive regulation of receptor internalization |
|  |  |  | GO:0033233 | 1.83E-03 | regulation of protein sumoylation |
|  |  |  | GO:0006264 | 5.67E-03 | mitochondrial DNA replication |
|  |  |  | GO:0010876 | 9.50E-03 | lipid localization |
|  |  |  | GO:0002097 | 9.53E-03 | tRNA wobble base modification |
|  |  | plum1 | GO:0008105 | 1.48E-04 | asymmetric protein localization |
|  |  |  | GO:0006468 | 2.76E-04 | protein phosphorylation |
|  |  |  | GO:0006355 | 3.40E-03 | regulation of transcription |
|  |  |  | GO:0002693 | 7.39E-03 | positive regulation of cellular extravasation |
|  |  | paleturquoise | GO:0006355 | 1.32E-04 | regulation of transcription |
|  |  |  | GO:0000122 | 1.83E-03 | negative regulation of transcription from RNA polymerase II promoter |
|  |  |  | GO:0042787 | 2.85E-03 | protein ubiquitination involved in ubiquitin-dependent protein catabolic process |
|  |  |  | GO:1903209 | 8.21E-03 | positive regulation of oxidative stress-induced cell death |
|  |  |  | GO:0080182 | 9.63E-03 | histone H3-K4 trimethylation |
|  |  | grey60 | GO:0051895 | 8.31E-04 | negative regulation of focal adhesion assembly |
|  |  |  | GO:0006418 | 3.73E-03 | tRNA aminoacylation for protein translation |
|  |  |  | GO:0007044 | 5.67E-03 | cell-substrate junction assembly |
|  |  |  | GO:0072431 | 9.17E-03 | signal transduction involved in mitotic G1 DNA damage checkpoint |
|  |  | green | GO:0071229 | 1.98E-03 | cellular response to acid chemical |
|  |  |  | GO:0048278 | 2.08E-03 | vesicle docking |
|  |  |  | GO:0070208 | 2.08E-03 | protein heterotrimerization |
|  |  |  | GO:1903053 | 3.41E-03 | regulation of extracellular matrix organization |
|  |  |  | GO:0099518 | 4.27E-03 | vesicle cytoskeletal trafficking |

| **Group** | **Time** | **Module** | **GO.ID** | ***p*-value** | **Term** |
| --- | --- | --- | --- | --- | --- |
| LRV1 | 8h | blue2 | GO:0030488 | 9.12E-05 | tRNA methylation |
|  |  |  | GO:0006544 | 1.85E-03 | glycine metabolic process |
|  |  |  | GO:0018126 | 5.47E-03 | protein hydroxylation |
|  |  |  | GO:0007005 | 7.68E-03 | mitochondrion organization |
|  |  |  | GO:0006355 | 7.71E-03 | regulation of transcription |
|  |  | coral3 | GO:0009062 | 6.58E-05 | fatty acid catabolic process |
|  |  |  | GO:0002683 | 4.10E-04 | negative regulation of immune system process |
|  |  |  | GO:0044270 | 8.94E-04 | cellular nitrogen compound catabolic process |
|  |  |  | GO:0045087 | 2.74E-03 | innate immune response |
|  |  |  | GO:0050728 | 3.18E-03 | negative regulation of inflammatory response |
|  |  | firebrick3 | GO:0070129 | 3.30E-04 | regulation of mitochondrial translation |
|  |  |  | GO:0006418 | 2.29E-03 | tRNA aminoacylation for protein translation |
|  |  |  | GO:0017004 | 4.42E-03 | cytochrome complex assembly |
|  |  |  | GO:0006390 | 7.06E-03 | transcription from mitochondrial promoter |
|  |  | navajowhite1 | GO:0090630 | 3.12E-03 | activation of GTPase activity |
|  |  |  | GO:0042445 | 3.98E-03 | hormone metabolic process |
|  |  |  | GO:0015914 | 4.84E-03 | phospholipid transport |
|  |  |  | GO:0007029 | 5.72E-03 | endoplasmic reticulum organization |
|  |  |  | GO:0036124 | 6.69E-03 | histone H3-K9 trimethylation |
|  |  | lavenderblush3 | GO:2000104 | 4.76E-04 | negative regulation of DNA-dependent DNA replication |
|  |  |  | GO:0045944 | 7.24E-03 | positive regulation of transcription from RNA polymerase II promoter |
|  |  |  | GO:2000050 | 9.15E-03 | regulation of non-canonical Wnt signaling pathway |
|  |  | plum | GO:0006086 | 1.96E-03 | acetyl-CoA biosynthetic process from pyruvate |
|  |  |  | GO:0043407 | 2.05E-03 | negative regulation of MAP kinase activity |
|  |  |  | GO:0050766 | 2.83E-03 | positive regulation of phagocytosis |
|  |  |  | GO:0033198 | 3.36E-03 | response to ATP |
|  |  |  | GO:0002675 | 6.45E-03 | positive regulation of acute inflammatory response |

| **Group** | **Time** | **Module** | **GO.ID** | ***p*-value** | **Term** |
| --- | --- | --- | --- | --- | --- |
| LRV1 | 24h | black | GO:0071805 | 9.24E-04 | potassium ion transmembrane transport |
|  |  |  | GO:0006165 | 2.17E-03 | nucleoside diphosphate phosphorylation |
|  |  |  | GO:0051181 | 2.99E-03 | cofactor transport |
|  |  |  | GO:0006910 | 3.90E-03 | phagocytosis |
|  |  |  | GO:0070542 | 3.90E-03 | response to fatty acid |
|  |  | skyblue3 | GO:0030488 | 1.18E-03 | tRNA methylation |
|  |  |  | GO:0032008 | 2.57E-03 | positive regulation of TOR signaling |
|  |  |  | GO:0032890 | 6.94E-03 | regulation of organic acid transport |
|  |  |  | GO:0046112 | 6.94E-03 | nucleobase biosynthetic process |
|  |  | lightsteelblue1 | GO:0006412 | 1.21E-06 | translation |
|  |  |  | GO:0000469 | 2.18E-06 | cleavage involved in rRNA processing |
|  |  |  | GO:0006103 | 3.32E-04 | 2-oxoglutarate metabolic process |
|  |  |  | GO:0043650 | 3.70E-03 | dicarboxylic acid biosynthetic process |
|  |  |  | GO:0016571 | 4.48E-03 | histone methylation |
|  |  | blue | GO:0055114 | 4.51E-06 | oxidation-reduction process |
|  |  |  | GO:0005975 | 1.11E-04 | carbohydrate metabolic process |
|  |  |  | GO:2000679 | 1.59E-03 | positive regulation of transcription regulatory region DNA binding |
|  |  |  | GO:0032259 | 3.12E-03 | methylation |
|  |  |  | GO:0006091 | 3.56E-03 | generation of precursor metabolites and energy |
|  |  | brown | GO:0032755 | 1.42E-07 | positive regulation of interleukin-6 production |
|  |  |  | GO:0032735 | 2.21E-06 | positive regulation of interleukin-12 production |
|  |  |  | GO:0032760 | 5.16E-06 | positive regulation of tumor necrosis factor production |
|  |  |  | GO:0070374 | 1.68E-05 | positive regulation of ERK1 and ERK2 cascade |
|  |  |  | GO:0042108 | 2.12E-05 | positive regulation of cytokine biosynthetic process |
|  |  | darkgrey | GO:0032968 | 9.93E-04 | positive regulation of transcription elongation from RNA polymerase II promoter |
|  |  |  | GO:0048024 | 2.25E-03 | regulation of mRNA splicing |
|  |  |  | GO:0051865 | 3.64E-03 | protein autoubiquitination |
|  |  |  | GO:0010390 | 6.65E-03 | histone monoubiquitination |
|  |  |  | GO:0009070 | 6.73E-03 | serine family amino acid biosynthetic process |
|  |  | red | GO:0000122 | 2.35E-04 | negative regulation of transcription from RNA polymerase II promoter |
|  |  |  | GO:0032722 | 5.23E-04 | positive regulation of chemokine production |
|  |  |  | GO:2000060 | 2.83E-03 | positive regulation of protein ubiquitination involved in ubiquitin-dependent protein catabolic process |
|  |  |  | GO:0032755 | 4.38E-03 | positive regulation of interleukin-6 production |
|  |  |  | GO:0042346 | 5.88E-03 | positive regulation of NF-kappaB import into nucleus |
|  |  | tan | GO:0061418 | 9.89E-04 | regulation of transcription from RNA polymerase II promoter in response to hypoxia |
|  |  |  | GO:0042107 | 1.03E-03 | cytokine metabolic process |
|  |  |  | GO:0033235 | 1.67E-03 | positive regulation of protein sumoylation |
|  |  |  | GO:0006355 | 4.63E-03 | regulation of transcription |
|  |  |  | GO:0007178 | 8.63E-03 | transmembrane receptor protein serine/threonine kinase signaling pathway |
|  |  | darkslateblue | GO:0033539 | 6.50E-06 | fatty acid beta-oxidation using acyl-CoA dehydrogenase |
|  |  |  | GO:0055114 | 2.16E-04 | oxidation-reduction process |
|  |  |  | GO:0044550 | 2.13E-03 | secondary metabolite biosynthetic process |
|  |  |  | GO:0055088 | 2.70E-03 | lipid homeostasis |
|  |  |  | GO:0006739 | 3.56E-03 | NADP metabolic process |

| **Group** | **Time** | **Module** | **GO.ID** | ***p*-value** | **Term** |
| --- | --- | --- | --- | --- | --- |
| Exacerbatory | 8h | green | GO:0006298 | 8.05E-04 | mismatch repair |
|  |  |  | GO:0032774 | 1.02E-03 | RNA biosynthetic process |
|  |  |  | GO:0051310 | 2.17E-03 | metaphase plate congression |
|  |  |  | GO:0006767 | 2.29E-03 | water-soluble vitamin metabolic process |
|  |  |  | GO:0009395 | 3.09E-03 | phospholipid catabolic process |
|  |  | orangered4 | GO:0046185 | 2.40E-04 | aldehyde catabolic process |
|  |  |  | GO:0006089 | 7.53E-04 | lactate metabolic process |
|  |  |  | GO:0055114 | 1.99E-03 | oxidation-reduction process |
|  |  |  | GO:0008652 | 4.91E-03 | cellular amino acid biosynthetic process |
|  |  |  | GO:0009084 | 6.93E-03 | glutamine family amino acid biosynthetic process |
|  |  | coral1 | GO:0006468 | 3.70E-04 | protein phosphorylation |
|  |  |  | GO:0007250 | 4.39E-04 | activation of NF-kappaB-inducing kinase activity |
|  |  |  | GO:2000637 | 4.39E-04 | positive regulation of gene silencing by miRNA |
|  |  |  | GO:0035329 | 6.52E-04 | hippo signaling |
|  |  |  | GO:0044827 | 6.77E-04 | modulation by host of viral genome replication |
|  |  | darkseagreen4 | GO:0007080 | 1.27E-04 | mitotic metaphase plate congression |
|  |  |  | GO:0000920 | 4.34E-04 | cell separation after cytokinesis |
|  |  |  | GO:0015986 | 9.17E-04 | ATP synthesis coupled proton transport |
|  |  |  | GO:0090502 | 1.66E-03 | RNA phosphodiester bond hydrolysis, endonucleolytic |
|  |  |  | GO:1901673 | 2.25E-03 | regulation of mitotic spindle assembly |
|  |  | bisque4 | GO:0051092 | 1.21E-04 | positive regulation of NF-kappaB transcription factor activity |
|  |  |  | GO:0001836 | 1.49E-04 | release of cytochrome c from mitochondria |
|  |  |  | GO:0050718 | 1.51E-04 | positive regulation of interleukin-1 beta secretion |
|  |  |  | GO:0032729 | 2.11E-04 | positive regulation of interferon-gamma production |
|  |  |  | GO:0032755 | 2.13E-04 | positive regulation of interleukin-6 production |
|  |  | darkgrey | GO:0006111 | 2.14E-03 | regulation of gluconeogenesis |
|  |  |  | GO:0043409 | 3.86E-03 | negative regulation of MAPK cascade |
|  |  |  | GO:0045815 | 4.16E-03 | positive regulation of gene expression, epigenetic |
|  |  |  | GO:0031122 | 4.73E-03 | cytoplasmic microtubule organization |
|  |  |  | GO:0031498 | 5.10E-03 | chromatin disassembly |
|  |  | mediumpurple4 | GO:0051016 | 3.78E-04 | barbed-end actin filament capping |
|  |  |  | GO:1903337 | 3.78E-04 | positive regulation of vacuolar transport |
|  |  |  | GO:0032988 | 6.43E-03 | ribonucleoprotein complex disassembly |

| **Group** | **Time** | **Module** | **GO.ID** | ***p*-value** | **Term** |
| --- | --- | --- | --- | --- | --- |
| Exacerbatory | 24h | darkolivegreen | GO:2000104 | 4.04E-04 | negative regulation of DNA-dependent DNA replication |
|  |  |  | GO:0035023 | 7.29E-04 | regulation of Rho protein signal transduction |
|  |  |  | GO:0051292 | 5.68E-03 | nuclear pore complex assembly |
|  |  |  | GO:0051592 | 6.41E-03 | response to calcium ion |
|  |  |  | GO:0006978 | 6.90E-03 | DNA damage response, signal transduction by p53 class mediator resulting in transcription of p21 class mediator |
|  |  | pink | GO:2000637 | 2.86E-03 | positive regulation of gene silencing by miRNA |
|  |  |  | GO:0005980 | 3.65E-03 | glycogen catabolic process |
|  |  |  | GO:0006206 | 3.65E-03 | pyrimidine nucleobase metabolic process |
|  |  |  | GO:0044249 | 5.41E-03 | cellular biosynthetic process |
|  |  |  | GO:1904874 | 5.59E-03 | positive regulation of telomerase RNA localization to Cajal body |
|  |  | yellow | GO:0046040 | 1.13E-03 | IMP metabolic process |
|  |  |  | GO:0009396 | 1.70E-03 | folic acid-containing compound biosynthetic process |
|  |  |  | GO:0006355 | 6.45E-03 | regulation of transcription, DNA-templated |
|  |  | magenta | GO:1903312 | 4.01E-04 | negative regulation of mRNA metabolic process |
|  |  |  | GO:0000122 | 1.18E-03 | negative regulation of transcription from RNA polymerase II promoter |
|  |  |  | GO:0010039 | 1.31E-03 | response to iron ion |
|  |  |  | GO:0010759 | 2.32E-03 | positive regulation of macrophage chemotaxis |
|  |  |  | GO:0016556 | 2.32E-03 | mRNA modification |
|  |  | brown4 | GO:1901800 | 1.10E-03 | positive regulation of proteasomal protein catabolic process |
|  |  |  | GO:0010561 | 2.92E-03 | negative regulation of glycoprotein biosynthetic process |
|  |  |  | GO:0043032 | 2.92E-03 | positive regulation of macrophage activation |
|  |  |  | GO:0040008 | 5.00E-03 | regulation of growth |
|  |  |  | GO:0051491 | 9.65E-03 | positive regulation of filopodium assembly |
|  |  | ivory | GO:0015914 | 1.84E-04 | phospholipid transport |
|  |  | turquoise | GO:0035458 | 1.59E-13 | cellular response to interferon-beta |
|  |  |  | GO:0051607 | 5.89E-12 | defense response to virus |
|  |  |  | GO:0071346 | 2.29E-11 | cellular response to interferon-gamma |
|  |  |  | GO:0045087 | 5.79E-08 | innate immune response |
|  |  |  | GO:0042832 | 3.71E-05 | defense response to protozoan |
