# Supplementary material for: Dissection of the macrophage response towards infection by the Leishmania-viral endosymbiont duo and dynamics of the type I interferon response
Source: Front Cell Infect Microbiol. 2022 Aug 4;12:941888. doi: 10.3389/fcimb.2022.941888 (PMC9386148; doi:10.3389/fcimb.2022.941888)
Supplement: Supplementary file 1 [file DataSheet_1.zip › Data Sheet 1/Supplementary Material/Table S3.docx]

**Table S3. Examples of main GO terms of modules in WT + *Ifnar^-/-^* analysis (biological process “BP” category and *p*-value < 0.01) at 8 and 24 hours post-infection.**

| **Time** | **Module** | **GO.ID** | ***p*-value** | **Term** |
| --- | --- | --- | --- | --- |
| 8h | greenyellow | GO:0051607 | 8.73E-15 | defense response to virus |
|  |  | GO:0071346 | 1.63E-12 | cellular response to interferon-gamma |
|  |  | GO:0045087 | 2.04E-11 | innate immune response |
|  |  | GO:0035458 | 2.23E-10 | cellular response to interferon-beta |
|  |  | GO:0042832 | 8.85E-10 | defense response to protozoan |
|  |  | GO:0045071 | 2.11E-08 | negative regulation of viral genome replication |
|  |  | GO:0034341 | 5.30E-07 | response to interferon-gamma |
|  |  | GO:0070374 | 6.30E-07 | positive regulation of ERK1 and ERK2 cascade |
|  |  | GO:0050729 | 1.20E-06 | positive regulation of inflammatory response |
|  |  | GO:0032735 | 4.63E-06 | positive regulation of interleukin-12 production |
|  |  | GO:0006925 | 7.82E-06 | inflammatory cell apoptotic process |
|  |  | GO:0032722 | 1.06E-05 | positive regulation of chemokine production |
|  |  | GO:0032693 | 1.28E-05 | negative regulation of interleukin-10 production |
|  |  | GO:0032729 | 1.64E-05 | positive regulation of interferon-gamma production |
|  |  | GO:0002720 | 1.93E-05 | positive regulation of cytokine production involved in immune response |
|  |  | GO:0032755 | 2.79E-05 | positive regulation of interleukin-6 production |
|  |  | GO:0045824 | 4.75E-05 | negative regulation of innate immune response |
|  |  | GO:0034121 | 4.77E-05 | regulation of toll-like receptor signaling pathway |
|  |  | GO:0060337 | 4.80E-05 | type I interferon signaling pathway |
|  |  | GO:0035455 | 4.84E-05 | response to interferon-alpha |
|  |  | GO:0002374 | 5.52E-05 | cytokine secretion involved in immune response |
|  |  | GO:0042510 | 5.52E-05 | regulation of tyrosine phosphorylation of Stat1 protein |
|  |  | GO:0050718 | 7.52E-05 | positive regulation of interleukin-1 beta secretion |
|  |  | GO:0002833 | 8.05E-05 | positive regulation of response to biotic stimulus |
|  |  | GO:0045429 | 8.05E-05 | positive regulation of nitric oxide biosynthetic process |
|  |  | GO:0032760 | 8.92E-05 | positive regulation of tumor necrosis factor production |
|  |  | GO:0032728 | 1.37E-04 | positive regulation of interferon-beta production |
|  |  | GO:0034097 | 1.50E-04 | response to cytokine |
|  |  | GO:0002429 | 1.59E-04 | immune response-activating cell surface receptor signaling pathway |
|  |  | GO:0002474 | 1.62E-04 | antigen processing and presentation of peptide antigen via MHC class I |
|  |  | GO:0032660 | 1.66E-04 | regulation of interleukin-17 production |
|  |  | GO:0002544 | 1.68E-04 | chronic inflammatory response |
|  |  | GO:0050728 | 1.84E-04 | negative regulation of inflammatory response |
|  |  | GO:0032088 | 1.95E-04 | negative regulation of NF-kappaB transcription factor activity |
|  |  | GO:0071347 | 1.96E-04 | cellular response to interleukin-1 |
|  | mediumpurple3 | GO:0009168 | 6.74E-06 | purine ribonucleoside monophosphate biosynthetic process |
|  |  | GO:0008033 | 8.07E-05 | tRNA processing |
|  |  | GO:0001522 | 9.75E-05 | pseudouridine synthesis |
|  |  | GO:0046040 | 2.59E-04 | IMP metabolic process |
|  |  | GO:0046033 | 6.10E-04 | AMP metabolic process |
|  |  | GO:0009152 | 8.99E-04 | purine ribonucleotide biosynthetic process |
|  |  | GO:0000959 | 1.18E-03 | mitochondrial RNA metabolic process |
|  |  | GO:0032259 | 1.44E-03 | methylation |
|  |  | GO:0006418 | 2.13E-03 | tRNA aminoacylation for protein translation |
|  |  | GO:0006139 | 2.67E-03 | nucleobase-containing compound metabolic process |
|  |  | GO:0046129 | 3.20E-03 | purine ribonucleoside biosynthetic process |
|  |  | GO:0000470 | 3.96E-03 | maturation of LSU-rRNA |
|  |  | GO:0006767 | 4.09E-03 | water-soluble vitamin metabolic process |
|  |  | GO:0051298 | 4.53E-03 | centrosome duplication |
|  |  | GO:0071426 | 4.55E-03 | ribonucleoprotein complex export from nucleus |
|  |  | GO:0030042 | 4.59E-03 | actin filament depolymerization |
|  |  | GO:0043966 | 6.06E-03 | histone H3 acetylation |
|  |  | GO:0000077 | 6.37E-03 | DNA damage checkpoint |
|  |  | GO:0007005 | 6.91E-03 | mitochondrion organization |
|  |  | GO:0010998 | 7.74E-03 | regulation of translational initiation by eIF2 alpha phosphorylation |
|  |  | GO:0045745 | 7.74E-03 | positive regulation of G-protein coupled receptor protein signaling pathway |
|  |  | GO:0046112 | 7.74E-03 | nucleobase biosynthetic process |
|  |  | GO:0018193 | 8.31E-03 | peptidyl-amino acid modification |
|  |  | GO:0009117 | 8.48E-03 | nucleotide metabolic process |
|  |  | GO:0030490 | 8.66E-03 | maturation of SSU-rRNA |
|  |  | GO:0055114 | 8.95E-03 | oxidation-reduction process |
|  |  | GO:0006364 | 9.90E-03 | rRNA processing |
| 24h | thistle1 | GO:0051607 | 7.51E-22 | defense response to virus |
|  |  | GO:0045087 | 2.31E-15 | innate immune response |
|  |  | GO:0035458 | 1.44E-12 | cellular response to interferon-beta |
|  |  | GO:0071346 | 7.74E-11 | cellular response to interferon-gamma |
|  |  | GO:0035455 | 1.52E-10 | response to interferon-alpha |
|  |  | GO:0045071 | 7.17E-10 | negative regulation of viral genome replication |
|  |  | GO:0002474 | 8.03E-07 | antigen processing and presentation of peptide antigen via MHC class I |
|  |  | GO:0042832 | 1.06E-06 | defense response to protozoan |
|  |  | GO:0060337 | 3.12E-06 | type I interferon signaling pathway |
|  |  | GO:0044406 | 2.16E-05 | adhesion of symbiont to host |
|  |  | GO:0034341 | 8.82E-05 | response to interferon-gamma |
|  |  | GO:0006955 | 1.22E-04 | immune response |
|  |  | GO:0032760 | 1.58E-04 | positive regulation of tumor necrosis factor production |
|  |  | GO:0035456 | 2.80E-04 | response to interferon-beta |
|  |  | GO:0032103 | 5.51E-04 | positive regulation of response to external stimulus |
|  | thistle2 | GO:0032755 | 5.29E-08 | positive regulation of interleukin-6 production |
|  |  | GO:0070374 | 3.88E-07 | positive regulation of ERK1 and ERK2 cascade |
|  |  | GO:0032760 | 4.02E-07 | positive regulation of tumor necrosis factor production |
|  |  | GO:0042108 | 8.64E-07 | positive regulation of cytokine biosynthetic process |
|  |  | GO:0045408 | 1.09E-05 | regulation of interleukin-6 biosynthetic process |
|  |  | GO:0032735 | 2.69E-05 | positive regulation of interleukin-12 production |
|  |  | GO:0051092 | 3.54E-05 | positive regulation of NF-kappaB transcription factor activity |
|  |  | GO:0032722 | 7.37E-05 | positive regulation of chemokine production |
|  |  | GO:0043123 | 7.58E-05 | positive regulation of I-kappaB kinase/NF-kappaB signaling |
|  |  | GO:0008360 | 1.14E-04 | regulation of cell shape |
|  |  | GO:0002833 | 1.21E-04 | positive regulation of response to biotic stimulus |
|  |  | GO:0051770 | 1.25E-04 | positive regulation of nitric-oxide synthase biosynthetic process |
|  |  | GO:0034612 | 1.86E-04 | response to tumor necrosis factor |
|  |  | GO:0032729 | 1.87E-04 | positive regulation of interferon-gamma production |
|  |  | GO:0042510 | 1.98E-04 | regulation of tyrosine phosphorylation of Stat1 protein |
|  | lightsteelblue1 | GO:0055114 | 1.49E-06 | oxidation-reduction process |
|  |  | GO:0006633 | 1.96E-05 | fatty acid biosynthetic process |
|  |  | GO:0033539 | 5.28E-05 | fatty acid beta-oxidation using acyl-CoA dehydrogenase |
|  |  | GO:0009396 | 1.73E-04 | folic acid-containing compound biosynthetic process |
|  |  | GO:0006636 | 3.21E-04 | unsaturated fatty acid biosynthetic process |
|  |  | GO:0006749 | 5.18E-04 | glutathione metabolic process |
|  |  | GO:0022898 | 7.61E-04 | regulation of transmembrane transporter activity |
|  |  | GO:0009168 | 9.59E-04 | purine ribonucleoside monophosphate biosynthetic process |
|  |  | GO:0018195 | 1.06E-03 | peptidyl-arginine modification |
|  |  | GO:1901264 | 1.35E-03 | carbohydrate derivative transport |
|  |  | GO:1990267 | 1.36E-03 | response to transition metal nanoparticle |
|  |  | GO:0006144 | 1.37E-03 | purine nucleobase metabolic process |
|  |  | GO:0006364 | 1.64E-03 | rRNA processing |
|  |  | GO:0009152 | 1.66E-03 | purine ribonucleotide biosynthetic process |
|  |  | GO:0046033 | 1.79E-03 | AMP metabolic process |
|  | magenta | GO:2000050 | 8.03E-04 | regulation of non-canonical Wnt signaling pathway |
|  |  | GO:0032508 | 1.42E-03 | DNA duplex unwinding |
|  |  | GO:0042994 | 2.16E-03 | cytoplasmic sequestering of transcription factor |
|  |  | GO:0051480 | 3.86E-03 | regulation of cytosolic calcium ion concentration |
|  |  | GO:0007188 | 4.55E-03 | adenylate cyclase-modulating G-protein coupled receptor signaling pathway |
|  |  | GO:0006399 | 6.56E-03 | tRNA metabolic process |
|  |  | GO:0010389 | 6.60E-03 | regulation of G2/M transition of mitotic cell cycle |
|  |  | GO:0001522 | 6.70E-03 | pseudouridine synthesis |
|  |  | GO:0070588 | 6.85E-03 | calcium ion transmembrane transport |
|  | tan | GO:0032088 | 1.60E-03 | negative regulation of NF-kappaB transcription factor activity |
|  |  | GO:0051603 | 2.33E-03 | proteolysis involved in cellular protein catabolic process |
|  |  | GO:0006511 | 2.41E-03 | ubiquitin-dependent protein catabolic process |
|  |  | GO:0097581 | 2.66E-03 | lamellipodium organization |
|  |  | GO:0030433 | 3.78E-03 | ER-associated ubiquitin-dependent protein catabolic process |
|  |  | GO:0006873 | 4.05E-03 | cellular ion homeostasis |
|  |  | GO:0001836 | 4.58E-03 | release of cytochrome c from mitochondria |
|  |  | GO:0019430 | 6.04E-03 | removal of superoxide radicals |
|  |  | GO:0042026 | 6.04E-03 | protein refolding |
|  |  | GO:1902117 | 6.12E-03 | positive regulation of organelle assembly |
|  |  | GO:0098771 | 6.27E-03 | inorganic ion homeostasis |
|  |  | GO:0016226 | 7.73E-03 | iron-sulfur cluster assembly |
|  |  | GO:0000186 | 8.03E-03 | activation of MAPKK activity |
|  |  | GO:0006904 | 8.03E-03 | vesicle docking involved in exocytosis |
|  |  | GO:0055081 | 8.03E-03 | anion homeostasis |
|  | darkmagenta | GO:0002544 | 6.02E-04 | chronic inflammatory response |
|  |  | GO:0050729 | 1.05E-03 | positive regulation of inflammatory response |
|  |  | GO:0002534 | 1.51E-03 | cytokine production involved in inflammatory response |
|  |  | GO:0051954 | 2.20E-03 | positive regulation of amine transport |
|  |  | GO:0010575 | 2.84E-03 | positive regulation of vascular endothelial growth factor production |
|  |  | GO:0070498 | 2.84E-03 | interleukin-1-mediated signaling pathway |
|  |  | GO:0010467 | 4.27E-03 | gene expression |
|  |  | GO:0042107 | 4.34E-03 | cytokine metabolic process |
|  |  | GO:0002190 | 4.39E-03 | cap-independent translational initiation |
|  |  | GO:0009072 | 5.74E-03 | aromatic amino acid family metabolic process |
|  |  | GO:0042402 | 5.74E-03 | cellular biogenic amine catabolic process |
|  |  | GO:0006955 | 7.52E-03 | immune response |
|  |  | GO:0061418 | 7.66E-03 | regulation of transcription from RNA polymerase II promoter in response to hypoxia |
|  |  | GO:0010972 | 8.61E-03 | negative regulation of G2/M transition of mitotic cell cycle |
|  |  | GO:0000122 | 8.85E-03 | negative regulation of transcription from RNA polymerase II promoter |
|  | lightyellow | GO:0050707 | 1.47E-03 | regulation of cytokine secretion |
|  |  | GO:0006355 | 2.54E-03 | regulation of transcription, DNA-templated |
|  |  | GO:0050858 | 2.74E-03 | negative regulation of antigen receptor-mediated signaling pathway |
|  |  | GO:0051769 | 2.74E-03 | regulation of nitric-oxide synthase biosynthetic process |
|  |  | GO:0007252 | 3.12E-03 | I-kappaB phosphorylation |
|  |  | GO:0002755 | 4.65E-03 | MyD88-dependent toll-like receptor signaling pathway |
|  |  | GO:0035329 | 4.65E-03 | hippo signaling |
|  |  | GO:0001817 | 6.36E-03 | regulation of cytokine production |
|  |  | GO:0000902 | 6.63E-03 | cell morphogenesis |
|  |  | GO:0032689 | 7.66E-03 | negative regulation of interferon-gamma production |
