# Supplementary material for: Dissection of the macrophage response towards infection by the Leishmania-viral endosymbiont duo and dynamics of the type I interferon response
Source: Front Cell Infect Microbiol. 2022 Aug 4;12:941888. doi: 10.3389/fcimb.2022.941888 (PMC9386148; doi:10.3389/fcimb.2022.941888)
Supplement: Supplementary file 1 [file DataSheet_1.zip › Data Sheet 1/Supplementary Material/Table S4.docx]

**Table S4. Non-exhaustive list of Interferon-Stimulated Genes (ISGs) and their membership to the modules at 8 and 24 hours post-infection in WT + *Ifnar^-/-^* analysis.** The corresponding values of closeness centrality (CC) at 8h (8h_CC) and 24 hours (24h_CC) post-infection of each gene are also indicated.

| **Genes** | **8h_modules** | **8h_CC** | **24h_modules** | **24h_CC** |
| --- | --- | --- | --- | --- |
| 9930111J21Rik1 | greenyellow | 0.677710706 | thistle1 | 0.309286215 |
| Acod1 | greenyellow | 0.62964858 | tan | 0.325357699 |
| Adar | greenyellow | 0.709093626 | thistle1 | 0.314869779 |
| Aim2 | greenyellow | 0.724730879 | tan | 0.323484356 |
| Apobec3 | greenyellow | 0.691665426 | thistle1 | 0.318826766 |
| Axl | greenyellow | 0.705385704 | thistle1 | 0.301434653 |
| Bst2 | greenyellow | 0.695752055 | thistle1 | 0.313035454 |
| Cactin | firebrick4 | 0.637401557 | white | 0.356984884 |
| Ccl2 | greenyellow | 0.681374687 | thistle2 | 0.320167634 |
| Ccl3 | greenyellow | 0.678546669 | thistle2 | 0.335750626 |
| Ccl5 | greenyellow | 0.675602113 | tan | 0.311600075 |
| Cd74 | thistle3 | 0.584727288 | grey | 0.188370757 |
| Cdc34 | floralwhite | 0.544089662 | plum1 | 0.370887883 |
| Cdc37 | purple | 0.370887883 | lightcyan | 0.360234825 |
| Ciita | midnightblue | 0.577769494 | purple | 0.274596077 |
| Cnot7 | greenyellow | 0.663734261 | thistle2 | 0.233935634 |
| Cxcl9 | greenyellow | 0.691348574 | thistle1 | 0.319896722 |
| Cxcl10 | greenyellow | 0.71831677 | thistle1 | 0.318018749 |
| Cxcl11 | greenyellow | 0.692390073 | thistle2 | 0.325969737 |
| Dcst1 |  |  |  |  |
| Ddit4 | firebrick4 | 0.57722023 | magenta | 0.304162586 |
| Ddx41 | orangered4 | 0.591225395 | lightcyan1 | 0.333468511 |
| Ddx58 | greenyellow | 0.670342608 | thistle1 | 0.309881106 |
| Ddx60 | greenyellow | 0.700284855 | thistle1 | 0.309868811 |
| Eif2ak2 | greenyellow | 0.66599076 | thistle1 | 0.309640064 |
| F830016B08Rik | greenyellow | 0.700497467 | thistle1 | 0.309443468 |
| Fadd | palevioletred3 | 0.569016562 | midnightblue | 0.303715013 |
| Gas6 | mediumpurple3 | 0.669913574 | magenta | 0.341944445 |
| Gbp1 |  |  |  |  |
| Gbp2 | greenyellow | 0.70006321 | thistle1 | 0.320877118 |
| Gbp2b | greenyellow | 0.680119178 | thistle1 | 0.307129235 |
| Gbp3 | greenyellow | 0.307129235 | thistle1 | 0.320692529 |
| Gbp6 | greenyellow | 0.677102875 | thistle1 | 0.314895954 |
| Gbp7 | greenyellow | 0.680944021 | thistle1 | 0.319290978 |
| Gm11772 | greenyellow | 0.665262848 | orange | 0.264557478 |
| Gm12185 | greenyellow | 0.718395083 | thistle2 | 0.319851596 |
| Gm45717 |  |  |  |  |
| Gm4841 | greenyellow | 0.690653991 | thistle2 | 0.320924256 |
| Gm4951 | greenyellow | 0.695867855 | thistle1 | 0.306010406 |
| Gm5431 | greenyellow | 0.692554297 | thistle1 | 0.308509473 |
| Hla-cd70 |  |  |  |  |
| Hpse | greenyellow | 0.667386587 | grey | 0.19520474 |
| Htra2 | darkseagreen3 | 0.535800428 | darkgreen | 0.368418309 |
| Ifi6 |  |  |  |  |
| Ifi16 |  |  |  |  |
| Ifi27 | greenyellow | 0.618723521 | greenyellow | 0.333553985 |
| Ifi30 | indianred4 | 0.522459527 | cyan | 0.360223172 |
| Ifi44l |  |  |  |  |
| Ifi47 | greenyellow | 0.707204874 | thistle1 | 0.309014432 |
| Ifi202a |  |  |  |  |
| Ifi202b | blue2 | 0.602186644 |  |  |
| Ifi203 | greenyellow | 0.602186644 | thistle1 | 0.307434953 |
| Ifi204 | greenyellow | 0.704845891 | thistle1 | 0.306936518 |
| Ifi205 | greenyellow | 0.709929109 | thistle1 | 0.314948539 |
| Ifi206 |  |  |  |  |
| Ifi207 |  |  |  |  |
| Ifi208 |  |  |  |  |
| Ifi209 |  |  |  |  |
| Ifi211 |  |  |  |  |
| Ifi213 |  |  |  |  |
| Ifi214 |  |  |  |  |
| Ifih1 | greenyellow | 0.66077177 | thistle1 | 0.315469589 |
| Ifit1 | greenyellow | 0.675452673 | thistle1 | 0.307586178 |
| Ifit2 | greenyellow | 0.659804246 | thistle1 | 0.307553094 |
| Ifit3 | greenyellow | 0.651872111 | thistle1 | 0.303163708 |
| Ifit5 |  |  |  |  |
| Ifitm1 | plum1 | 0.60745028 | grey | <0.05 |
| Ifitm2 | plum1 | 0.534780159 | red | 0.244481321 |
| Ifitm3 | greenyellow | 0.702776942 | thistle1 | 0.308924344 |
| Ifitm6 | plum1 | 0.645331965 | thistle1 | 0.306561788 |
| Ifitm7 | thistle3 | 0.566889877 | lightcyan1 | 0.312122542 |
| Ifna1 |  |  |  |  |
| Ifna4 |  |  |  |  |
| Ifnar1 | greenyellow | 0.661177695 | thistle1 | 0.296544192 |
| Ifnar2 | greenyellow | 0.668559999 | thistle1 | 0.300302274 |
| Ifnb1 | greenyellow | 0.697678172 | thistle2 | 0.326966604 |
| Ifnw1 |  |  |  |  |
| Igtp | greenyellow | 0.716358702 | thistle1 | 0.319928587 |
| Iigp1 | greenyellow | 0.718814612 | thistle1 | 0.310533317 |
| Ikbke | greenyellow | 0.679205022 | thistle2 | 0.318077416 |
| Il15 | greenyellow | 0.688524681 | thistle2 | 0.321496159 |
| Irak1 | white | 0.675855967 | orangered4 | 0.371675296 |
| Irf1 | greenyellow | 0.716208571 | thistle2 | 0.322385233 |
| Irf2 | greenyellow | 0.670728695 | thistle1 | 0.313779123 |
| Irf3 | mediumpurple3 | 0.646040384 | lightcyan | 0.331876958 |
| Irf7 | greenyellow | 0.69544704 | thistle1 | 0.30631063 |
| Irf9 | greenyellow | 0.692325118 | thistle1 | 0.302841463 |
| Irgc1 |  |  |  |  |
| Irgm1 | greenyellow | 0.680471403 | thistle1 | 0.313236145 |
| Irgm2 | greenyellow | 0.711214905 | thistle1 | 0.313739663 |
| Isg15 | greenyellow | 0.656109013 | thistle1 | 0.314333205 |
| Isg20 | greenyellow | 0.720923688 | thistle1 | 0.320945356 |
| Jak2 | greenyellow | 0.682167713 | thistle2 | 0.329264555 |
| Lsm14a | greenyellow | 0.714554566 | tan | 0.31771948 |
| Map3k14 | firebrick4 | 0.631691174 | lightcyan | 0.307069626 |
| Mavs | mediumpurple3 | 0.669224095 | purple | 0.296065673 |
| Mb21d1 | greenyellow | 0.640200367 | thistle1 | 0.311645565 |
| Mettl3 | mediumpurple3 | 0.640439254 | darkmagenta | 0.327283886 |
| Mmp12 | mediumpurple3 | 0.602106499 | darkorange | 0.377503283 |
| Mndal | greenyellow | 0.715156826 | thistle1 | 0.31086056 |
| Mov10 | greenyellow | 0.727573826 | thistle1 | 0.319709682 |
| Mul1 | greenyellow | 0.666146373 | thistle2 | 0.344976316 |
| Mx1 | greenyellow | 0.660402464 | thistle1 | 0.306968654 |
| Mx2 | greenyellow | 0.67269932 | thistle1 | 0.3081083 |
| Myc | mediumpurple3 | 0.648235285 | lightsteelblue1 | 0.351793297 |
| Myd88 | greenyellow | 0.726654499 | thistle2 | 0.323360463 |
| Ms4a4a | greenyellow | 0.627104467 | thistle1 | 0.298478508 |
| Nampt | blue2 | 0.654636138 | tan | 0.333155931 |
| Nlrc5 | greenyellow | 0.676146619 | thistle1 | 0.314309616 |
| Nos2 | greenyellow | 0.718010372 | thistle2 | 0.329476672 |
| Nt5c3 | greenyellow | 0.714326737 | thistle1 | 0.319653904 |
| Oas1 |  |  |  |  |
| Oas1a | greenyellow | 0.68594447 | thistle1 | 0.297473497 |
| Oas1c | greenyellow | 0.697016104 | thistle1 | 0.30895665 |
| Oas1d |  |  |  |  |
| Oas1e |  |  |  |  |
| Oas1f |  |  |  |  |
| Oas1g | greenyellow | 0.645314123 | thistle1 | 0.299735726 |
| Oas1h |  |  |  |  |
| Oas1x |  |  |  |  |
| Oas1y |  |  |  |  |
| Oas1z |  |  |  |  |
| Oas2 | greenyellow | 0.684193878 | thistle1 | 0.299555959 |
| Oas3 | greenyellow | 0.684597761 | thistle1 | 0.303277648 |
| Oasl1 | greenyellow | 0.675195321 | thistle1 | 0.307361444 |
| Oasl2 | greenyellow | 0.630754479 | thistle1 | 0.29506569 |
| P2ry6 | green | 0.610529417 | magenta | 0.322931527 |
| Pde12 | firebrick4 | 0.658582847 | purple | 0.237708933 |
| Phf15 |  |  |  |  |
| Pml | greenyellow | 0.71646845 | thistle1 | 0.319567925 |
| Pnpt1 | greenyellow | 0.714675517 | thistle1 | 0.314454556 |
| Ptpn2 | greenyellow | 0.691668399 | thistle2 | 0.330419527 |
| Rnf185 | maroon | 0.581307946 | plum1 | 0.344063251 |
| Ro60 |  |  |  |  |
| Rsad2 | greenyellow | 0.653327218 | thistle1 | 0.310365009 |
| Rtp4 | greenyellow | 0.618019317 | thistle1 | 0.296651413 |
| Samhd1 | greenyellow | 0.730556399 | thistle1 | 0.316775156 |
| Slc15a3 | greenyellow | 0.699322625 | tan | 0.350773258 |
| Slc25a28 | greenyellow | 0.654478151 | thistle2 | 0.334034964 |
| Socs1 | greenyellow | 0.66945684 | thistle1 | 0.320351792 |
| Socs3 | greenyellow | 0.699970877 | thistle2 | 0.334396672 |
| Ssbp3 | lightgreen | 0.644259712 | grey | 0.174859783 |
| Star |  |  |  |  |
| Stat1 | greenyellow | 0.707918086 | thistle1 | 0.310796686 |
| Stat2 | greenyellow | 0.698258781 | thistle1 | 0.311709908 |
| Sting1 |  |  |  |  |
| Sun2 | antiquewhite2 | 0.559054922 | plum1 | 0.371903469 |
| Tbk1 | greenyellow | 0.674852635 | thistle2 | 0.335988751 |
| Tgtp1 | greenyellow | 0.727295071 | thistle1 | 0.322809265 |
| Tgtp2 | greenyellow | 0.699869623 | thistle1 | 0.309152225 |
| Ticam1 | maroon | 0.561681098 | thistle2 | 0.318243628 |
| Timp1 | thistle3 | 0.591715586 | darkmagenta | 0.335506114 |
| Tlr3 | greenyellow | 0.686263483 | thistle1 | 0.320419226 |
| Tlr7 | greenyellow | 0.705586301 | lightyellow | 0.302395761 |
| Tpr | darkolivegreen | 0.63867609 | darkmagenta | 0.270208816 |
| Trex1 |  |  |  |  |
| Trim2 | mediumpurple3 | 0.664494135 | lightsteelblue1 | 0.350112061 |
| Trim5 | darkmagenta | 0.512206171 | thistle1 | 0.293466797 |
| Trim6 | greenyellow | 0.67715279 | tan | 0.327570179 |
| Trim21 | greenyellow | 0.72821181 | thistle1 | 0.320683701 |
| Trim25 | greenyellow | 0.677293629 | thistle1 | 0.326392238 |
| Trim34 |  |  |  |  |
| Trim36 | palevioletred3 | 0.553450033 | greenyellow | 0.345557714 |
| Trim41 | lightgreen | 0.603175043 | orange | 0.21992559 |
| Trim56 | darkolivegreen | 0.662639869 | thistle2 | 0.32081785 |
| Ttll12 | mediumpurple3 | 0.651587465 | orangered4 | 0.375375729 |
| Ube2g2 | firebrick4 | 0.585684038 | plum1 | 0.380195367 |
| Ube2k | blue2 | 0.590995905 | violet | 0.322297699 |
| Usp27x | lightcoral | 0.514603233 | grey | <0.05 |
| Usp29 |  |  |  |  |
| Wnt5a |  |  |  |  |
| Ythdf2 | orange | 0.613225642 | lightyellow | 0.264115958 |
| Ythdf3 | ivory | 0.527539226 | grey | 0.170071468 |
| Zbp1 | greenyellow | 0.655720944 | thistle1 | 0.303701482 |
| Zc3hav1 | greenyellow | 0.699802303 | thistle1 | 0.327911426 |
